# Differential functional organization of amygdala-medial prefrontal cortex networks in macaque and human

**DOI:** 10.1038/s42003-024-05918-y

**Published:** 2024-03-05

**Authors:** Camille Giacometti, Delphine Autran-Clavagnier, Audrey Dureux, Laura Viñales, Franck Lamberton, Emmanuel Procyk, Charles R. E. Wilson, Céline Amiez, Fadila Hadj-Bouziane

**Affiliations:** 1 Univ Lyon, Université Lyon 1, Inserm, Stem Cell and Brain Research Institute U1208, 69500 Bron, France; 2 Inovarion, 75005 Paris, France; 3 Integrative Multisensory Perception Action & Cognition Team (ImpAct), INSERM U1028, CNRS UMR5292, Lyon Neuroscience Research Center (CRNL); Université Lyon 1, 69500 Bron, France; 4 La Structure Fédérative de Recherche Santé Lyon-Est, CNRS UAR 3453, INSERM US7, Lyon 1 University, 69008 Lyon, France; 5 Centre d’Etude et de Recherche Multimodal et Pluridisciplinaire en Imagerie du Vivant (CERMEP), 69677 Bron, France

**Keywords:** Neuroscience, Evolution

## Abstract

Over the course of evolution, the amygdala (AMG) and medial frontal cortex (mPFC) network, involved in behavioral adaptation, underwent structural changes in the old-world monkey and human lineages. Yet, whether and how the functional organization of this network differs remains poorly understood. Using resting-state functional magnetic resonance imagery, we show that the functional connectivity (FC) between AMG nuclei and mPFC regions differs between humans and awake macaques. In humans, the AMG-mPFC FC displays U-shaped pattern along the corpus callosum: a positive FC with the ventromedial prefrontal (vmPFC) and anterior cingulate cortex (ACC), a negative FC with the anterior mid-cingulate cortex (MCC), and a positive FC with the posterior MCC. Conversely, in macaques, the negative FC shifted more ventrally at the junction between the vmPFC and the ACC. The functional organization divergence of AMG-mPFC network between humans and macaques might help understanding behavioral adaptation abilities differences in their respective socio-ecological niches.

## Abstract (web summary)

A resting-state fMRI study in both humans and macaques suggest that the functional connectivity pattern between amygdala nuclei and the medial prefrontal cortex present similarities and differences that may explain differential behavioral adaptation.

## Introduction

In the face of uncertain environments, one must quickly detect salient information and adapt in consequence. Animals constantly monitor their surroundings (peer interactions, resource availability, danger, etc.), while also considering information related to their own internal state (emotional, motivational and physiological)^1^. A growing number of studies converge toward a critical role of the network formed by the medial prefrontal frontal cortex (mPFC) and the amygdala (AMG) in behavioral adaptation ability^2–8^. Both regions are highly heterogeneous. The AMG is a complex structure composed of several interconnected nuclei^2,9^. The lateral nucleus (LA) is the main entry of sensory inputs, the basolateral nucleus (BL) and the basomedial nucleus (BM) are gating information from higher cognitive processes regions (e.g., mPFC), and the central nucleus (CE), is tightly connected with the autonomous system^10^. Within the mPFC, the ventro-medial PFC (vmPFC) and the anterior cingulate cortex (ACC) are involved in environmental stimulus valuation in the light of current internal states, while the mid-cingulate cortex (both its anterior -aMCC- and posterior -pMCC- part) is involved in outcome- and action-based decision monitoring^1,11–16^.

Although the regions composing this network find their homologs in macaques and humans, they present structural differences that might result from the influence of environmental and social factors relative to the respective ecological niche of each species. First, the AMG is 10 times larger in humans compared to macaques due in particular to a larger expansion of LA nucleus^17–21^. Second, although the macaque mPFC displays all the sulcal precursors of the human mPFC, the region interfacing with vmPFC and MCC (which contains ACC) expanded in humans^22^. The present paper aims at identifying whether and how these structural changes affect the functional coupling within the AMG-mPFC network.

By means of resting state functional MRI, a powerful cross-species reproducible method^23–26^, we compared the functional connectivity (FC) pattern between the various AMG nuclei and mPFC regions in both awake humans (*n* = 20) and awake macaques (*n* = 3) as we have shown that anaesthesia alters FC within the frontal cortex^27^. Results show that, in humans, the AMG-mPFC FC displays a rostro-caudal U-shaped pattern along the corpus callosum: positive FC with vmPFC and ACC, negative FC with anterior MCC, and positive FC with posterior MCC. By contrast, although a U-shape FC organization is observed in macaques, the negative FC shifted more ventrally between the AMG and the region located at the junction between vmPFC and ACC. We also show that this FC pattern is driven by all AMG nuclei in both species, with the exception of the CE in humans. Altogether, these results highlight an anatomo-functional organization of the AMG-mPFC network divergence in the cercopithecoid monkeys and human lineages.

## Results

**Fig. 1:**
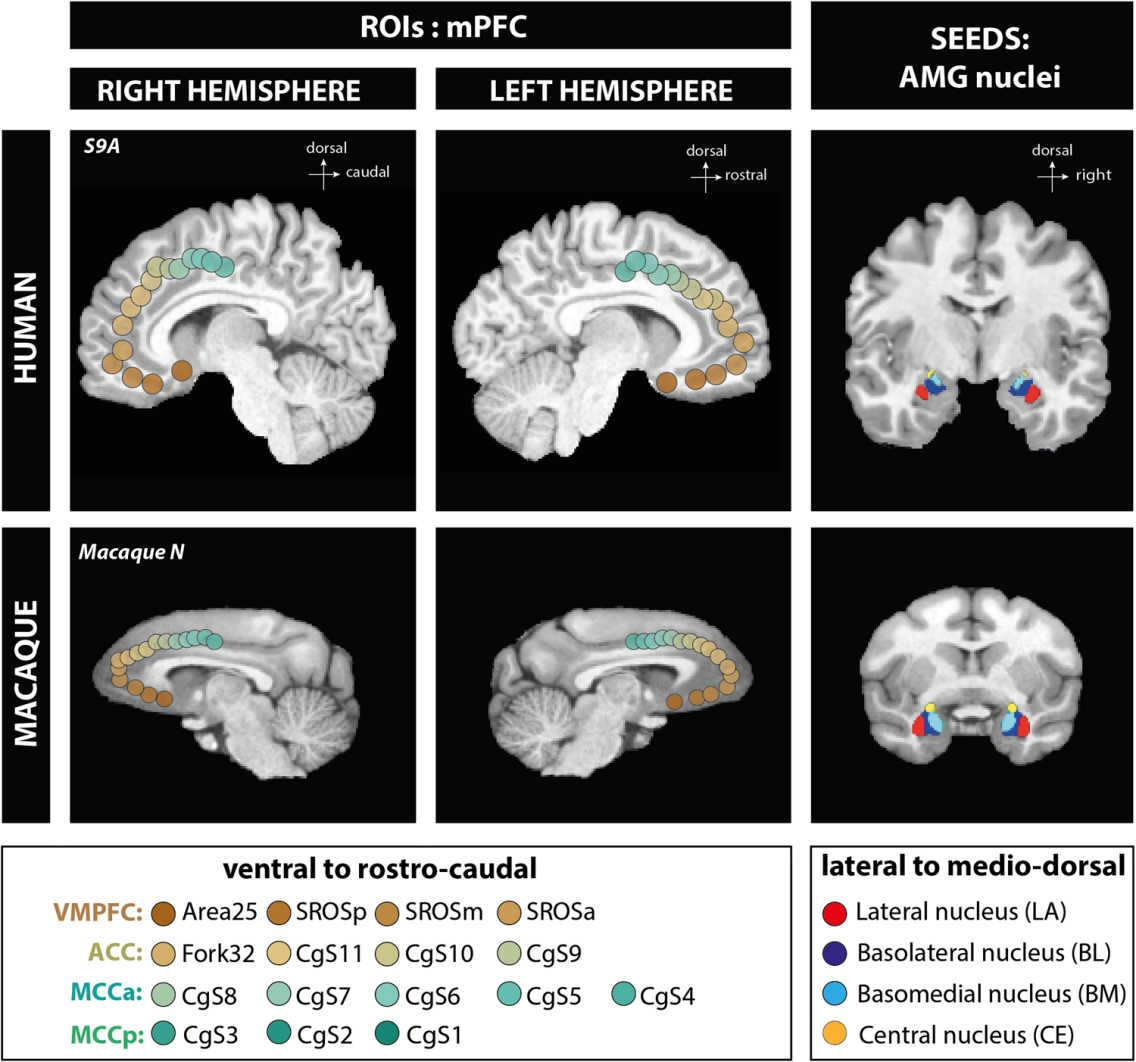
Amygdala nuclei SEEDs and medial prefrontal cortex ROIs localization in human and macaque in the right and left hemispheres. SEEDs and ROIs are displayed on the top panel for humans and on the bottom panel for macaques. On the left panel, mPFC ROIs localization on mid-sagittal brain sections in both hemispheres. The 16 ROIs are color-coded from brown to seagreen gradient in the ventro-dorsocaudal axis along the corpus callosum: vmPFC: Area25, SROSp, SROSm, SROSa; ACC: Fork32, CgS11, CgS10, CgS9; aMCC: CgS8, CgS7, CgS6, CgS5, CgS4; pMCC: CgS3, CgS2 and CgS1. On the right panel, AMG 4 main nuclei, extracted from Tyszka an Pauli (2016) atlas for humans and SARM atlas for macaques, illustrated on coronal sections. Lateral (LA) in red, basolateral (BL) in dark blue, basomedial (BM) in cyan and central (CE) in yellow.

In both humans and macaques, we assessed FC between (1) the atlas-based parcellation of the 4 main AMG nuclei (CE, BL, BM, and LA)^28,29^, and (2) a fine-grained parcellation of the mPFC (16 ROIs) based on anatomical sulcal landmarks^22^ (Fig. 1). The mPFC ROIs were spheres covering (1) the vmPFC (4 ROIs: subgenual Area 25, 3 ROIs in the Superior Rostral Sulcus, the posterior –SROSp–, medial –SROSm–, anterior –SROSa– part), (2) the ACC (4 ROIs on a rostrocaudal axis: Fork32 - part of cytoarchitectonic area 32 located just anterior to the fork formed by the suprarostral and the sus-orbitalis sulcus, CgS11, CgS10 and CgS9), (3) the aMCC (5 ROIs on a rostrocaudal axis in the cingulate sulcus: CgS8, CgS7, CgS6, CgS5, CgS4), and (4) the pMCC (3 ROIS on a rostrocaudal axis: CgS3, CgS2, CgS1). Note that results presented in the main text correspond to the AMG-mPFC FC pattern observed in the right hemisphere. The FC pattern observed in the left hemisphere is displayed in Supplementary Information (Figs. S1 and S2).

### Functional connectivity within the AMG-mPFC network in humans

**Fig. 2:**
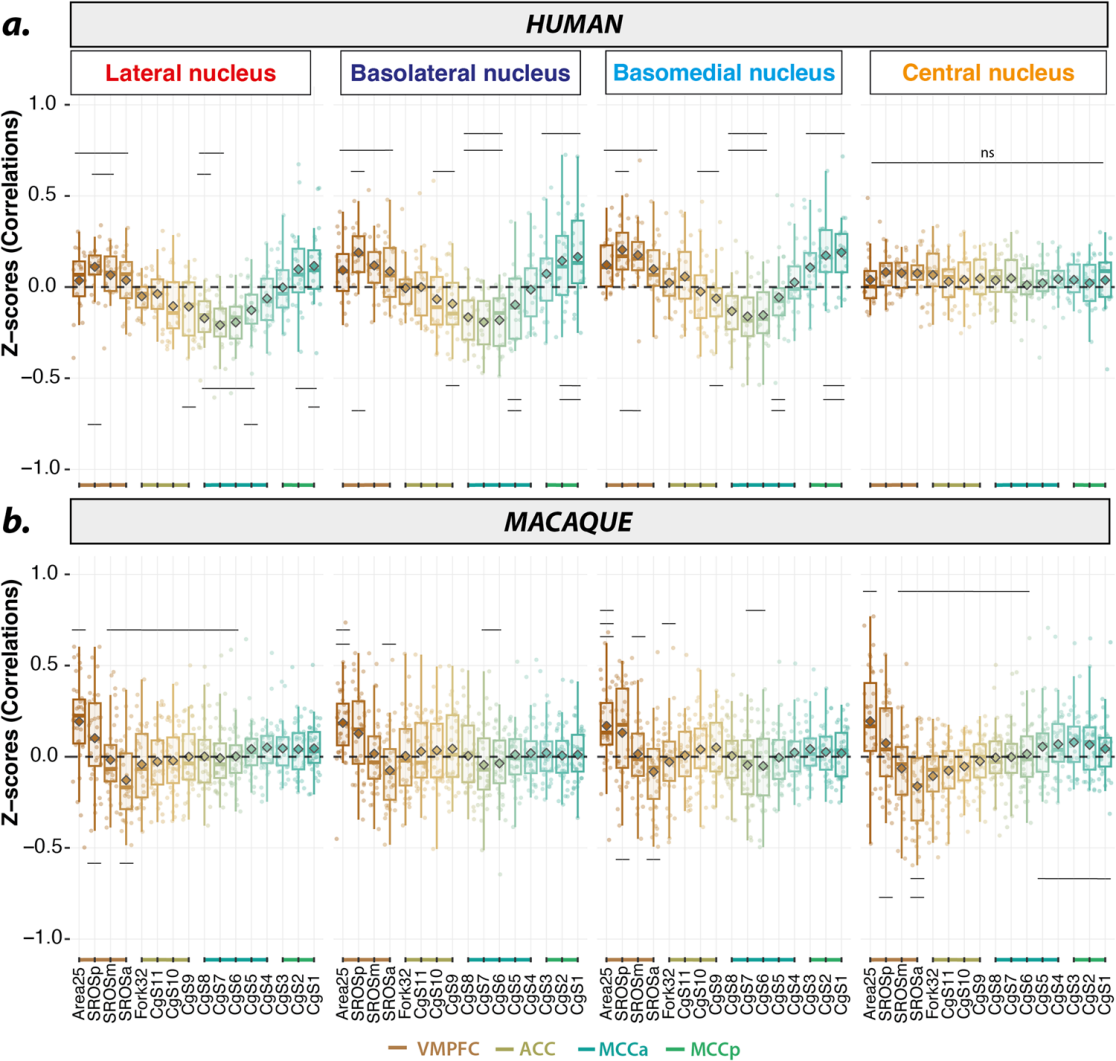
Functional connectivity pattern between AMG nuclei and mPFC ROIs in humans and macaques. Boxplots display correlation strength (z-scores) between each AMG nuclei (SEEDs) and the 16 mPFC ROIs ordered from ventral-to-dorso-caudal for each species: **a** humans *n* = 20 and (**b**) macaques, *n* = 3 * 12 runs. In each boxplot, the lower and upper hinges of the box correspond to the first and third quartiles respectively. Upper and lower whiskers extent from their corresponding hinges to the largest and lowest value respectively define as 1.5 x of the interquartile range. Diamonds overlaid in dark gray on the boxplots represent the mean. Individual data points are also represented for each boxplot. The horizontal black lines represent significant pairwise results within SEEDs associated with FDR corrected *p* values. **a** Humans. Results show a U-shape functional pattern for LA, BL and BM but not for CE: mPFC ventral ROIs (vmPFC) present positive z-score values, then z-scores decrease, reaching a negative peak in aMCC (ROIs CgS7-CgS6) and z-scores increase back to positive values in pMCC. GLMM: significant effect of SEEDS (*F* = 19.103, df = 3, *p* = 4.197e-12), ROIs (*F* = 28.805, df = 15, *p* < 2.2e-16) and their interactions (*F* = 2782, df = 45, *p* = 6.531e-09). Pairwise significant comparison p.value range from 2.633645e-09 to 0.04610722 with df = 1197. **b**. Macaques. The 4 AMG SEEDs present a similar functional pattern: more ventral vmPFC ROIs present positive z-scores, then z-scores decrease reaching a negative peak in dorsal vmPFC ROIs (ROI SROSa) and z-scores increase back towards positive value in MCC ROIs. GLMM: significant effect of ROIs (F(15,2205) = 22.5703, *p* < 2e-16), no effect of SEEDs (F(3,2205) = 0.8367, *p* = 0.47364) and a trend for SEEDs *ROIs interactions (F(45,2205) = 1.2860, *p* = 0.09731). Pairwise significant comparison *p* value range from 1.676072e-08 to 0.02322724 with df = 2205.

The correlation strengths between AMG nuclei (LA, BL, BM and CE SEEDs) and mPFC ROIs are displayed on boxplots in Fig. 2a Statistical analysis using General Linear Mixed Model -GLMM- with “SEEDs” and “ROIs” as fixed factors (see Methods) revealed a significant main effect of SEEDs (F(3,1197) = 19.103, *p* = 4.197e-12), ROIs (F(15,1197) = 28.805, *p* = < 2.2e-16) and an interaction between SEEDs and ROIs (F(45,1197) = 2.782, *p* = 6.531e-09) pointing toward a differential FC pattern between AMG nuclei and mPFC ROIs (see Table S1 for a complete description of the statistical results). Specifically, the FC between CE and mPFC ROIs at rest is close to zero and does not present any specific pattern. By contrast, the BL, BM and LA SEEDs present a U-shaped FC pattern with the various ROIs of the mPFC along a ventro-dorsocaudal axis. They display positive correlations with vmPFC ROIs (i.e., from Area25 to SROSa for LA and BL, and from Area25 to CgS11 for BM), negative correlations with ACC/aMCC ROIs (from Fork32 to CgS4/CgS3), and positive correlations with pMCC ROIs (CgS3, CgS2, CgS1). Importantly, the most negative FC in the U-shaped pattern is located within mPFC ROIs CgS6 to CgS8 -part of MCCa- for LA and BM and BL SEEDs with a peak at ROI CgS7 (pairwise post-hoc significant comparisons, *p* < 0.05 ranging from 2.633645e-09 to 0.04610722). These results are further confirmed by the SEED-ROI pairs correlation strength comparison to 0 significantly highlighting the negative curve along ROI peak CgS7 for LA, BL and BM and the positive correlation within vmPFC (see also Fig. S3 in Supplementary Information). Note that results in the left hemisphere are similar to those observed for the right hemisphere and are presented in Supplementary Information (Figs. S1, S2, and Table S1). To confirm that these correlation profiles did not depend on physical distance between SEEDs and ROIs, we calculated the Euclidean distances between the different SEEDs and ROIs (Figs. S4 and S5 for the right and left hemisphere, respectively). Results confirmed that the z-scores (displayed in Fig. 2a) do not strictly vary as a function of distance (Fig. S6).

### Functional connectivity within AMG-mPFC networks in awake macaques

Correlation strengths between AMG nuclei and mPFC ROIs are displayed on boxplots in Fig. 2b Contrary to humans, the GLMM analysis revealed no main effect of SEEDs (F(3,225) = 0.8367, *p* = 0.47364) nor any significant interaction between SEEDs and ROIs F(45,2225) = 1.2860, *p* = 0.09731 (see Methods, Supplementary Information and Table S1 for details). It however revealed a significant main effect of ROIs (F(15,2205) = 22.5703, *p* < 2e-16). Results point toward a similar FC pattern between all AMG nuclei and mPFC ROIs that fluctuates depending on mPFC ROIs. Two main differences in macaques compared to humans were identified: (1) the 4 AMG SEEDs, including CE, display a U-shape FC pattern with mPFC ROIs, and (2) macaques present a different U-shape FC pattern in which the negative FC relationship between all AMG nuclei with mPFC ROIs extended from ROIs SROSp to Fork32 -part of vmPFC- with a negative peak located at the level of ROI SROSa (Fig. 2, significant pairwise comparisons, p.values ranging from 1.676072e-08 to 0.02322724 and see also Fig. S3 showing SEED-ROI pairs displaying a correlation strength significantly different from 0 using one sample T.tests). Within vmPFC, the most ventro-caudal ROIs (i.e., ROI Area25 and SROSp) present a high positive correlation strength with all AMG nuclei similar to the one observed in humans (Figs. 2b and S3). In addition, in macaques, the FC between BM and BL AMG nuclei tends to display negative functional coupling with mPFC ROIs CgS6 to CgS7, i.e., with the aMCC region, although not significantly different from 0 in the right hemisphere (Fig. S3). Note that results in the left hemisphere are slightly different in macaques (Figs. S1, S2 and Table S1) with significant effects of the factor “SEEDs” and of the “SEEDs-ROIs” interaction, mostly driven by CE. Finally, as in humans, this gradient did not depend on mere physical distance as assessed with the Euclidean distance between each AMG nuclei and mPFC ROIs pairs for each subject (Figs. S4, S5 and S6). Of note, the FC pattern observed in awake macaque monkeys with rewarded ocular fixation (Fig. 2) is similar to that observed when monkeys do not perform ocular fixation and thus do not receive any rewards (Fig. S7). Note also that the connectivity profile between mPFC and AMG nuclei in the awake state was greatly reduced under anaesthesia (Isoflurane 1–1.5%) for the same 3 monkeys (Figs. S8 and S9).

### An FC shift between macaques and humans: a species-specific pattern?

**Fig. 3:**
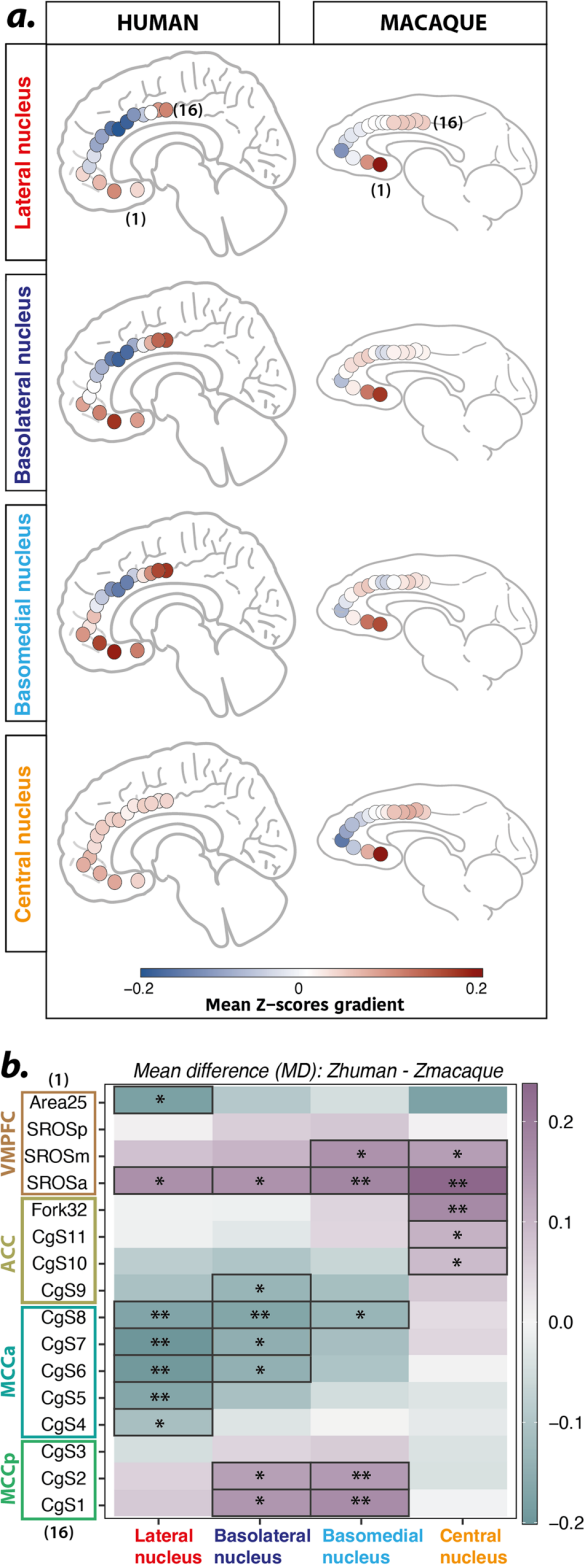
Comparison of functional connectivity between AMG nuclei and mPFC ROIs in macaque *versus* human. **a** Mean functional FC (expressed as z-scores) for each seed with mPFC ROIs in humans (left part) and macaques (right part) on mid-sagittal views. Mean z-scores values are displayed as a positive-to-negative gradient color-coded from red-to-blue. **b** Mean difference (MD) heatmap: z-scores_human_-zscores_macaque_ for each seed-ROI pair. MD is color-coded from pastel cyan to purple corresponding to negative and positive difference respectively. Two-sided Student test (df = 54) significant differences between species are highlighted: * for *p* < 0.05 and ** for *p* < 0.01 with *p* values ranging from 0.0017088 to 0.04646957. These results demonstrate two key differences between humans and monkeys: (1) a differential FC pattern of the CE nuclei with mPFC ROIs and (2) a differential functional coupling (positive versus negative) of mPFC ROIs with AMG nuclei, with a negative coupling in aMCC in humans and in vmPFC in macaques. These results suggest a ventral shift of the negative FC between macaques and humans.

In humans, the most negative FC was observed between CgS7 (within aMCC) and LA (−0.21 ± 0.16), BL (−0.19 ± 0.16), and BM (−0.16 ± 0.18) nuclei. In macaques, the most negative FC was observed more anteriorly: between SROSa and LA (−0.13 ± 0.20), BL (−0.07 ± 0.21), BM (−0.08 ± 0.21), and CE (−0.16 ± 0.23) nuclei. Thus, the negative FC peak, which triggers the U-shape FC gradient, differs critically between the 2 species: whereas it is located in aMCC (CgS7) in humans, it is located in the anteriormost part of vmPFC (SROSa) in macaques (Fig. 3a). Note that this differential functional topography between macaque and human is supported by an additional analysis assessing the FC of the whole AMG with the mPFC (see Fig. S9). Another main difference is the FC of CE that follows the same pattern as the other nuclei in macaques but not in humans (Fig. 3a). To further characterize these differences, we computed mean differences of correlation strength between each human AMG seed with mPFC ROIs (ordered from ventral-to-dorso-caudal) compared to their macaque homologs (Fig. 3b). Two-sided Student test results showed significant mean differences between humans and macaques regarding (1) FC between the aMCC region (CgS8 to CgS6) and both LA and BL, (2) FC between the ACC/aMCC limit (CgS9 and CgS8), and BM, BL, and LA (t(df = 54) and *p* < 0.05 ranging from from 0.0017088 to 0.04646957, Fig. 3b). These results confirmed a differential FC organization between humans and macaques characterized by a shift of the negative FC curve from aMCC in humans to vmPFC/ACC in macaques. In addition, it confirms a differential pattern of FC of the CE AMG nuclei between humans and monkeys (Fig. 3a, b).

## Discussion

The aim of this study was twofold: (1) to determine the functional connectivity pattern between AMG main nuclei (LA, BL, BM and CE) and mPFC regions, and (2) to identify whether and how this organization evolved between the cercopithecoid monkeys and human lineages since the split from their last common ancestor. By exploring intrinsic spontaneous low-frequency correlations in rs-fMRI signal, we show that whereas AMG activity is negatively correlated with aMCC activity in humans, it is negatively correlated with activity of the region located at the intersection between the vmPFC and the ACC in macaques. We also identified the contribution of all AMG nuclei in this pattern in both species, with the exception of the CE in humans (Fig. 2). These data first refine our knowledge on the complex functional dialogue between AMG and mPFC in humans^21,30–34^ by precisely seizing (i) a FC silhouette with a positive-to-negative transition area within the aMCC and (ii) the absence of contribution of the CE nucleus to this pattern. Second, it provides novel information of the AMG-mPFC dialogue in macaques by identifying (i) a shift of the positive-to-negative transition area to the vmPFC/ACC intersection region and (ii) the contribution of the CE nucleus to this pattern. Our study thus critically uncovers two key differences in the AMG-mPFC FC organization between humans and monkeys: an antero-posterior shift in the AMG dialogue with the mPFC from macaques to humans and a differential connectivity pattern of the CE nucleus, both suggesting a divergence between the two species.

Our results first revealed a differential functional connectivity organization between AMG nuclei and mPFC and behavioral significance in humans and macaques. To date, only a few studies have examined the functional interplay between AMG and mPFC in macaques using resting-state fMRI^33,35,36^. However, these studies did not capture the fine-grained organization of this interplay because of 2 main factors: (1) they considered the AMG as a whole and not the AMG nuclei separately, and (2) they have been carried out under anaesthesia, which has been shown to strongly affect frontal cortical FC^21,27^. The observed functional dialogue could be supported by the known structural connectivity in macaques^37–39^. Indeed, tract-tracing studies have shown that the most caudal part of vmPFC (Area25) and the MCC are densely connected to AMG nuclei, while the rostral part of vmPFC (SROSa) and the ACC share lesser anatomical connections with AMG nuclei. The presence of a U-shape FC pattern, characterized by negative functional coupling with AMG nuclei uncovered in our study, may therefore reflect the specific known structural connectivity between these regions^40^, featuring the existence of a transitional zone in the rostral part of vmPFC (SROSa) in macaques. By contrast, our results show that, in humans, this vmPFC region displays a positive functional coupling with 3 AMG nuclei (LA, BL and BM). This result may appear surprising given that MRI tractography studies have suggested that fiber tracts between the AMG and the mPFC seem to be preserved between humans and monkeys at the macroscopic level^41,42^. However, this latter finding should be taken cautiously^43,44^. Indeed, contrary to macaques, our knowledge of the detailed structural connectivity at the microscopic level in this network in humans is lacking, preventing direct comparisons between structural connectivity at the microscopic level and functional relationships. Importantly, the differences between humans and macaques observed in the present study find support in the known structural differences both in the mPFC and the AMG. First, the assessment of the evolution of the sulcal organization of the mPFC in the primate order has revealed that the only mPFC region that displays a strong evolution is the transition between vmPFC/ACC region^22^. This is precisely where we identified the main difference between species. Second, the total volume of AMG and its nuclei evolved in the primate order^17–21,45^: the largest expansion was found in the LA nucleus, occupying the major portion of the AMG in humans, compared to great apes^18,20^ and macaques^17,19^ where the BL nucleus presents the largest volume^17,20^. It is thus reasonable to suggest that with an increasing volume and neuron number in humans, the AMG might display more intricate connections with mPFC regions, resulting in a differential functional interplay between AMG nuclei and mPFC^46^. In human adults, the MCC is known to exert a strong top-down control onto the AMG^47^. Importantly, this top-down control is acquired during development. From childhood to adolescence and early adulthood, a shift from bottom-up (AMG to mPFC) to top-down regulatory processes has been described^48–51^. Indeed, AMG responses decrease concomitantly with the emergence of stronger top-down influences from mPFC during adolescence that further strengthen in adulthood compared to childhood in response to fearful faces^51^. This is in line with our findings in adult humans identifying negative FC between ACC/aMCC and AMG nuclei BM/LA/BL at rest (Figs. 2a and 3a). In adult rhesus macaques, this negative FC pattern was shifted ventrally in the vmPFC/ACC (Figs. 2b and 3a) for the 4 AMG nuclei, including the CE nucleus. Based on these results, it is reasonable to hypothesize that the source of top-down control might be differently balanced in humans and in macaques, and this shift might reflect differential regulatory processes in adaptive behaviors. Adult macaques *rhesus* are characterized by specific behavioral traits such as aggressiveness and impulsivity^52^ that are greatly reduced following AMG lesions^53–55^. It is thus reasonable to hypothesize that, compared to humans, a reduced top-down regulatory control exerted onto the AMG leads to higher AMG reactivity associated with higher emotional responsiveness in macaques. It is important to highlight that the identification of a differential fine-grained FC pattern in the AMG-mPFC network in macaques and humans could be unveiled thanks to the sulcal-based positioning of homologous mPFC ROIs in both species. Indeed, recent advances have revealed a remarkably similar sulcal mPFC and lateral PFC organization in the macaque and human brains that allows the identification of homologous regions^22,56^.

We also highlighted a differential contribution of the CE nucleus in the AMG-mPFC functional dialogue in humans and macaques. In humans, contrary to macaques, the FC of the CE nucleus at rest was close to zero and did not present any specific pattern with mPFC regions. Based on anatomical evidence, a differential functional dialogue of the CE on one hand and of BL/BM/LA on the other hand would be expected. First, during ontogeny, the CE does not originate from the same structure as BL/BM/LA, opposing a pallial versus a subpallial origins^57^. Second, these different developmental origins may thus explain their differential structural -and consequently functional- connections: contrary to BL/BM/LA, CE shares only very weak structural connections with the mPFC and is rather mostly connected to autonomic centers such as brainstem and hypothalamus^10^. Third, the CE nucleus is thought to be the most preserved AMG nucleus during evolution in terms of morphology (i.e., volume, neuron numbers etc.)^16^. However, the connectivity and function of the CE nucleus in the primate order may have evolved. Indeed, the CE nucleus is part of the extended amygdala, i.e., one of the main substrates for defensive behavior (i.e., avoidance-approach responses)^58^. It has also been shown to be susceptible to stressful environmental influences during development^59^ and involved in anxious and stress-related behaviors as its removal reduced stressed/anxious responses in macaques^54^. Importantly, in their specific ecological niches, humans and macaques do not face the same environmental challenges (e.g., less food availability issues, lack of predators in humans compared to macaques, etc.). Accordingly, macaques are constantly on high alert, balancing predator vigilance, within-group vigilance, and the need to access food^60^. Consequently, we hypothesize that the CE-mPFC FC pattern observed in macaques -as opposed to humans- may be driven by stronger bottom-up excitatory inputs (AMG to mPFC) and reduced top-down regulation from mPFC onto the AMG, stemming in particular from the expansion of the vmPFC/ACC region. This functional divergence between macaques and humans may relate to the inherent characteristics of their respective ecological niches. As the CE does not display direct connections with the mPFC^10^, its contribution to the AMG-mPFC FC in macaques may depend on its indirect functional connectivity, involving or not the autonomous centers.

As limitations, first, although our results display similar FC profiles in the AMG-mPFC network in macaques engaged in (i) an ocular fixation task in which they received rewards or (ii) not engaged in such a task (i.e., sleepy runs, see Figs. 2, 3 and S7), humans were by contrast engaged only in an ocular fixation task without receiving rewards. While our results in macaques suggest that the FC pattern in the AMG-mPFC network is not affected by the context of juice reward and ocular fixation, a final statement regarding any impact of the reward on this FC pattern would require the use of the exact same behavioral (i.e., adding reward in the human protocol or removing it in the macaque protocol) and MRI acquisition (i.e., using a coil allowing the use of multi-echo and multiband sequences in the macaque protocol) protocols in both humans and macaques. Second, one may hypothesize that the lack of contribution of the CE nucleus to the U-shape FC in the AMG-mPFC network in humans could be attributed to the limited number of voxels of this nucleus and/or, more generally, to the different numbers of voxels included in the SEEDs versus the ROIs. Indeed, in human brains, AMG SEEDs are smaller than the mPFC ROIs, but the 3 nuclei contributing equally to the U-shape FC pattern (LA, BL, BM) display different numbers of voxels. The number of voxels in the human CE nucleus is not significantly different from the BM nucleus (in the right hemisphere: 80 mm³ vs. 110 mm³, Table S2), the latest being extensively involved in the U-shape FC pattern. In addition, the correlation profile remained stable regardless of the number of voxels subsampled within the amygdala LA nucleus (Fig. S10). Finally, in macaque brains, with the exception of the CE nucleus, the volume and number of voxels in the AMG SEEDs are similar to those in the mPFC ROIs, strongly suggesting that voxel size does not significantly impact the FC pattern in the AMG-mPFC network (Table S3). Of note, the CE nucleus, i.e., which displays the smallest number of voxels, is the nucleus exhibiting the strongest contribution to the U-shape FC pattern in macaques.

**Fig. 4:**
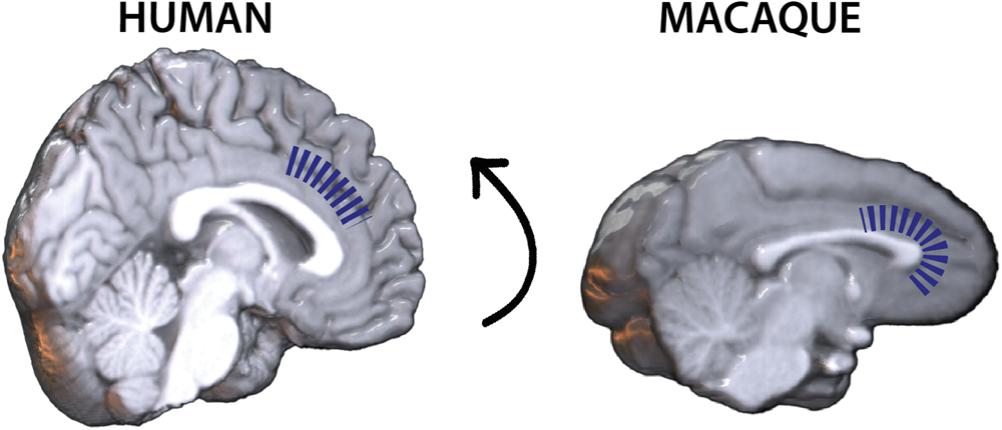
Schematical representation of the functional dialogue between AMG nuclei and mPFC regions in human and macaque. Blue dashed lines represent the extent of negative functional correlations between AMG nuclei and mPFC regions on structural brain images in human (right) and macaque (left). We identified a dorsal shift (represented by the arrow) in the functional gradient from vmPFC to aMCC for macaques and humans respectively, that might reflect differences governing bottom-up and top-down regulatory processes essential for flexible behavioral adaptation to the ecological niche.

To conclude, the present study identified a differential functional interplay between AMG nuclei and mPFC subregions between humans and macaques (see Summary in Fig. 4) that may reflect structural differences governing bottom-up and top-down regulatory processes in response to changes in internal and external milieu, thus triggering differential adaptive behaviors appropriately to their respective socio-ecological niche. Future studies employing fine-grained effective connectivity in both species may help better understand the complex functional interplay within this network at the heart of behavioral adaptation^51^ and identify whether and how the connectivity of the CE nucleus have evolved differently in the old-world monkeys and human lineages.

## Methods

### Participants

#### Humans

Twenty healthy subjects participated in the resting-state fMRI experiment (14 F and 6 M; age 25.6 ± 5.3). They all sign an informed consent form and also received monetary compensation at the end of the session. All ethical regulations relevant to human research participants were followed. The study was approved by a national ethics committee in biomedical research (Comité de Protection des Personnes (CPP) Sud-Est III, authorization ID: 2015-A00897-42 and 2018-A00405-50). It also received Clinical Trial Numbers (NCT03119909 and NCT03483233, see https://clinicaltrials.gov). Because the ventro-dorsal extent of the cingulate cortex in humans depends on the presence or not of a paracingulate sulcus (PCGS, presents in about 70% of subjects in at least one hemisphere^61^), we selected subjects based on this morphological feature in order to obtain a sample in which 50% of both left and right hemispheres presented a PCGS, and 50% did not.

#### Rhesus macaques

Three rhesus monkeys (*Macaca mulatta*) were included in the study (2 F: Monkeys C, 21 years old and N 9.5 years old and 1 M: Monkey L, 9.5 years old; weight 5–8 kg). Animals were maintained on a water and food regulation schedule, individually tailored to maintain a stable level of performance for each monkey. All procedures follow the guidelines of European Community on animal care (European Community Council, Directive No. 86–609, November 24, 1986) and were approved by French Animal Experimentation Ethics Committee #42 (CELYNE).

### Rs-fMRI data acquisition in humans

Scanning was performed on a 3 T Siemens Magnetom Prisma MRI Scanner (Siemens Healthcare, Germany). Details of the procedure can be found in Table 1.

Rs-fMRI runs lasted 10 min. Subjects were instructed to keep still and maintain fixation on a white cross presented at the center of the screen. Data were acquired with a T2* weighted multiband and multi-echo (ME) sequence: TR = 1500 ms, TE1 = 16.4 ms, TE2 = 37.59 ms, TE3 = 58.78 ms, voxel size = 2.5 mm^3^. We collected 1 runs of rs-fMRI (400 TRs) for each subject. An anatomical MRI was also obtained (see Table 1).

### Rs-fMRI data acquisition in macaques

Scanning was also performed on a 3 T Siemens Magnetom Prisma MRI Scanner (Siemens Healthcare, Germany, Table 1). Rs-fMRI runs lasted 13 min. Subjects were trained to maintain fixation on a white cross presented on the center of the screen in order to receive a liquid reward through the runs. Data were acquired with a T2* weighted gradient echo planar sequence: TR = 1800 ms, TE = 16.4 ms, voxel size = 1.8 mm^3^. We collected 12 runs of rs-fMRI (400 TRs/run) for each subject (Table 1 for details).

**Table 1:** MRI acquisition parameters for humans and awake rhesus macaques

MRI Acquisition parameters		
Species	Human	Rhesus macaque
Sample	*n* = 20	*n* = 3
MRI Scanner	3 T Siemens Magnetom Prisma	
rs-fMRI sequence: T2*-weighted gradient echo planar EPI images		
Slices	51	30
Spatial voxel resolution	2.5 mm^3^	1.8 mm^3^
Temporal resolution (TR)	1.5 s	1.8 s
Echo times (TE)	TE_1_ = 16.4 ms TE_2_ = 37.59 ms TE_3_ = 58.78 ms	TE = 27 ms
Volumes	400 vol/run	400 vol/run
Number of Runs	1/subject	12/subject
T1 weighted MPRAGE sequence: anatomical scans		
Slices	244	144
Spatial voxel resolution	0.8 mm^3^	0.5 mm^3^
Temporal resolution (TR)	3 s	3 s

#### Headpost surgical procedure

To limit head motion, macaque monkeys were head-fixed during MRI acquisition. They were first surgically implanted with a PEEK MR-compatible head post (Rogue Research, CA) under aseptic conditions. Animals were sedated prior to intubation (tiletamine and zolazepam, Zoletil 7 mg/kg) and then maintained under gas anaesthesia with a mixture of O2 and air (isoflurane 1–2%). After an incision of the skin along the skull midline, the head fixation device was positioned under stereotaxic guidance on the skull and maintained in place using ceramic sterile screws (Thomas RECORDING products) and acrylic dental cement (Palacos® Bone cements). Throughout the surgery, heart rate, respiration rate, blood pressure, expired CO_2_, and body temperature were continuously monitored. Analgesic and antibiotic treatment were administered for 5 days postoperatively and a recovery period of at least 1 month was observed after the surgery.

#### Experimental setup for awake macaque monkey

The setup is also detailed in ref. ^27^. Shortly, before the scanning session, macaques were trained head-fixed in a mock scanner mimicking the actual MRI environment in an MRI compatible plastic chair (Rogue Research). They were trained to fixate a central cross for long periods of time using positive reinforcement learning (juice-reward). During the scanning sessions, eye position was monitored using an eye-tracking system (Eyelink, SR research). The calibration procedure involved a central point and 4 additional points (up, down, left, right, 5° eccentricity), presented sequentially in the same plane as the fixation cross. Throughout the rs-fMRI sessions, monkeys were required to fixate a central cross on the screen (4 × 4°) in order to receive liquid reward through a plastic tube placed in their mouth. In the reward schedule and to promote long periods of fixation, the frequency of reward delivery increased as the duration of fixation increased^62^. The mean time with eyes open across runs was, respectively, 69%, 69%, and 84% for Monkeys L, N, and C. Within this time, the percentage of fixation varied from 36% to 69%, 2% to 58%, and 5% to 98% for Monkeys L, N, and C, respectively. During scanning sessions, we also collected several runs in which Monkey L (6 runs) and N (4 runs) did not perform ocular fixation (with eyes open or close/sleepy,) resulting in no rewards delivery. In our previous paper^27^, we showed that the juice reward associated with ocular fixation did not impact the FC pattern of frontal cortical networks. We found similar results within the AMG-mPFC network, suggesting stable FC within this network under different task conditions (Fig. S7, Supplementary Information).

#### Anaesthetized acquisition session

A high-resolution T1-weighted anatomical (MPRAGE, 0.5mm3 isotropic voxels, 144 slices, TR = 3000 ms, TE = 366 ms) was also acquired in a different session where macaques were maintained under anaesthesia. During this anaesthetized session, we also acquire resting-state functional runs for the 3 macaque monkeys. Briefly, monkeys were first injected with an anticholinergic agent decreasing salivary secretion (Robinul; 0.06 mg/kg). The animals were then anaesthetized 20 min later with an intramuscular injection of tiletamine and zolazepam (Zoletil; 7 mg/kg), intubated and ventilated with oxygen enriched air and 1–1.5% Isoflurane throughout the duration of the scan. Monkeys were placed in a sphinx position with their head maintained in an MRI-compatible stereotaxic frame (Kopf, CA, USA). Breathing volume and frequency were set based on the animal weight, body temperature was maintained using warm-air circulating blankets, and physiological parameters were monitored. The rs-fMRI acquisitions were performed 1h after first inhalation of isoflurane. Three receive  Siemens ring coils were used for the acquisition: 2 L11 on each side of the monkey’s head and 1 L7 Siemens above the monkey’s head. Rs-fMRI functional images were obtained with a T2*-weighted gradient echo planar images (EPI) sequence with the following parameters: for Monkeys L and N, TR = 1700 ms, TE = 30 ms, 25 slices, voxel size: 1.6 mm^3^ and for Monkey C, TR = 2000 ms, TE = 30 ms, 31 slices, voxel size: 1.8 mm^3^. We collected 6 runs for monkeys L and C and 5 runs for monkey N with 400 volumes per run. Results for anaesthetized monkeys state are displayed in Supplementary Information in Figs. S8 and S9.

### SEEDs and ROIs selections

The main goal of the rs-fMRI analyses was to investigate the FC pattern between AMG nuclei and mPFC in humans and macaques. Our analysis focuses on the ipsilateral functional connectivity of the 4 main AMG nuclei, Central (CE), Basolateral (BL), Basomedial (BM), and Lateral (LA), chosen as our seed regions, and 16 mPFC regions chosen as our ROIs located in the vmPFC, ACC and MCC. Location of SEEDs and ROIs are displayed on Fig. 1 for humans and macaque monkeys in both hemispheres. For both species, we also provide SEEDs and ROIs masks volume and number of voxels included in Supplementary Information (Table S2 for humans and Table S3 for macaques).

#### Amygdala SEEDs

The four main AMG nuclei masks were extracted from ref. ^29^ atlas for humans and from the Subcortical Atlas of the Rhesus Macaque (SARM) atlas for macaques^28^. LA is situated on the lateral part of the AMG complex and is ventrally and caudally bounded by the temporal horn of the lateral ventricle and laterally by temporal lobe white matter. BL is bounded laterally by LA. In humans, in the atlas^29^, the BL nucleus mask comprises both BL and paralaminar nucleus. Therefore, we also combined these nuclei in macaques. BM is located medially to BL. CE lies dorsally and caudally within the AMG complex.

#### Medial prefrontal Cortex ROIs

mPFC ROIs were precisely positioned based on local anatomical sulcal landmarks in both individual human and macaque subjects^20^. Indeed, the sulcal pattern in the mPFC is preserved in the primate order and allows to infer homologies between primate species^22,56^. Moreover, to account for differences in brain size across species, ROI dimensions were adjusted to a radius of 6 mm and 2.5 mm for humans and macaques, respectively (Fig. 1). Indeed, the antero-posterior extent of the human brain in the MNI template is 175 mm (https://www.bic.mni.mcgill.ca/ServicesAtlases/ICBM152NLin2009) and of the macaque brain in the NMT template is 72 mm (https://afni.nimh.nih.gov/pub/dist/doc/htmldoc/nonhuman/macaque_tempatl/template_nmtv2.html^63^,). The radius of each mPFC ROIs being 6 mm in humans, we thus used a radius of 2.5 mm in macaques to conserve the proportions (i.e., 6*72/175 = 2.5). Specifically, ROIs were positioned along the ventro-dorsocaudal axis of the corpus callosum (CC). In the ventral portion of mPFC below the corpus callosum, the vmPFC includes 4 ROIS: Area25 (localized in the Broadman area 25), SROSp, SROSm, and SROSa. SROS ROIs are named after the Superior Rostral Sulcus. The prefix p, m and a, respectively corresponding to posterior, medial and anterior part of SROS. Rostrally to the genu of the corpus callosum, the ACC includes 4 ROIs. Fork 32 located just in front to the fork situated at the rostral end of CGS formed by the supra-rostral sulcus (SU-ROS) and the supra-orbital sulcus (SOS), presumably occupied by area 32. It also includes several ROIs within the cingulate sulcus (CgS): CgS11, CgS10, and CgS9. Posteriorly to the genu of the genu of corpus callosum, the MCC includes 8 ROIs: CgS8, CgS7, CgS6, CgS5, CgS4 in the aMCC and CgS3, CgS2, CgS1 in the pMCC. In humans, cingulate ROIs cover both banks of the cingulate sulcus and the paracingulate sulcus (PCGS) if present. While not present in the macaque brains, a PCGS is present in 70% of subjects in at least one hemisphere in humans^22,61^. Note that when a PCGS was present, for a given ROI, two spheres were positioned on both CGS and PCGS and averaged to form one ROI (Supplementary Information, Figure S11: PCGS ROIs localization in both hemispheres).

### Neuroimaging data processing

Data analysis was performed using SPM12, AFNI^64^, FSL^65^ and R.

### Preprocessing

#### Humans

The first 5 volumes of each run were removed to allow for T1 equilibrium effects. Slice timing correction for multiband sequences was then applied and TEDANA package 56 was used to combine the 3 echo time series and to perform motion correction. The combined data is decomposed via, first, a principal component analysis (PCA) and second, an independent component analysis (ICA). TE-dependent components are classified as BOLD signal, while TE-independent components are classified as non-BOLD signal, and are discarded. For more information, please check TEDANA community page: https://zenodo.org/record/4509480#.YmEnNy8RqJ8^66–69^. Functional and anatomical images were then spatially normalized into standard MNI space.

#### Macaques

The first 5 volumes of each run were removed to allow for T1 equilibrium effects. First, we performed a slice timing correction using the time center of the volume as reference. The head motion correction was then applied using rigid body realignment. Then, images were skull-stripped using the bet tool from the FSL software (https://fsl.fmrib.ox.ac.uk/fsl/fslwiki/BET). Using the AFNI and FSL softwares, the segmentation of each brain of each session was performed on skull-stripped brains. To ensure optimized inter-session and inter-subject comparisons, both anatomical and functional images were registered in the NMT v2 template space^63^ to (1) ensure optimized inter-session and inter-subject comparisons and (2) use SARM atlas^28^ for AMG parcellation.

Note that for both species, the registration of individual macaque and human brains to their respective template has been carefully checked individually for each human and macaque monkey subject.

### Functional connectivity pattern analysis in humans and macaques

For both species, a temporal filtering was applied to extract the spontaneous slowly fluctuating brain activity (0.01–0.1 Hz). Linear regression was used to remove nuisance variables (the six parameter estimates for head motion, the cerebrospinal fluid and white matter signal from brain segmentation). A spatial smoothing with a 6-mm and a 4-mm full-width half maximum (FWHM) Gaussian kernel, for humans and macaques respectively, was applied to the output of the regression. For each subject in each run, each species, and each hemisphere, we computed the averaged correlation coefficient between the 4 AMG nuclei’s activity and the activity of each of the 16 mPFC ROIs using Pearson correlation scores. Those correlation scores were then normalized using the Fisher r-to-z transform formula.

### Statistics and reproducibility

#### Intra-species statistical analysis

In order to characterize the FC organization pattern for each seed and for each separately, we computed a global General Linear Mixed Model (GLMM, lsmeans package https://cran.r-project.org/web/packages/lsmeans/lsmeans.pdf). GLMM were built for each species and for each hemisphere separately with “SEEDs” and “ROIs” as main factor and “SUBJECT” as a random factor. In humans, “PCGS” factor was added as a random factor (1 | PCGS). In macaques, we added “RUN” as a random factor (1 | RUN). To account for inter-run variability for each subject, we added as random factor the effect of “RUN” within “SUBJECT”: (1 | SUBJECT:RUN). Main GLMM effects are displayed for each species in Table S1 (Supplementary Information). GLMM were followed by post-hoc pairwise comparisons to assess for any differences/similarities in correlation strength between each Seed-ROIs pair. In addition, and to better characterize the functional peaks observed in the previous results, each SEED-ROI pair correlation strength was compared to 0 using a one sample Student test for each species and in each hemisphere (Fig. S3). All *p* values were adjusted with False Discovery Rate (FDR) correction for multiple comparisons with an alpha level set to 0.05 for both humans and macaques. We also analyze AMG FC connectivity with mPFC ROIs by merging and averaging the 4 AMG nuclei SEEDs for each species. Results are displayed in Supplementary Information in Fig. S9. Note that we did not test for any inter-hemispheric differences given our small number of macaque subjects. Hence the statistics were carried out for each hemisphere separately in both humans and monkey to allow inter-species comparisons. We also calculated Euclidean distances (ED) as a measure of physical distance between each AMG seed and mPFC ROIs for both species. EDs were computed using the x, y and z coordinates for each subject in accordance with their local morphology (Figs. S4 and S5 for the right and left hemisphere respectively). Z-score values were further expressed as a function of ED to examine possible correlation linking physical distance and FC. Results are displayed in Supplementary Information (Fig. S6) and show that in both species the ED does not predict the Z-score values.

#### Inter-species comparison and statistical analysis

We computed the mean z-score for each Seed-ROI pair and displayed it as color-coded heatmaps on brain schemas. The red-blue gradient corresponds to positive-to-negative z-score values, respectively (Fig. 3a). To compare the AMG-mPFC FC patterns in both species, we computed the statistical mean difference (MD) between humans and macaques for each Seed-ROI pair and compared them with two-sided Student Test (Fig. 3b). Humans were used as the reference group. P.values were FDR corrected for multiple comparisons with an alpha level set to 0.05.

### Reporting summary

Further information on research design is available in the Nature Portfolio Reporting Summary linked to this article.

### Supplementary information

Supplementary Information

Description of Supplementary Materials

Supplementary Data 1

Supplementary Data 2

Reporting summary

## Data Availability

The authors declare that the numerical data table supporting the findings of this study are available as Supplementary Data: Data Table S1 for humans and Data Table S2 for macaques.

## Code availability

Codes are available upon reasonable request to the corresponding author.
