# Supplementary material for: Differential functional organization of amygdala-medial prefrontal cortex networks in macaque and human
Source: Commun Biol. 2024 Mar 5;7:269. doi: 10.1038/s42003-024-05918-y (PMC10914752; doi:10.1038/s42003-024-05918-y)
Supplement: Supplementary file 1 — Supplementary Information [file 42003_2024_5918_MOESM1_ESM.pdf]

Supplementary Information for:

Differential functional organization of amygdala-medial prefrontal cortex networks in macaque and human

Camille Giacometti[1†], Delphine Autran-Clavagnier[1,2], Audrey Dureux[2], Laura Viñales[1], Franck Lamberton[3,4], Emmanuel Procyk[1], Charles R.E. Wilson[1], Céline Amiez[1†*,] Fadila Hadj-Bouziane[5†*]

[1] Univ Lyon, Université Lyon 1, Inserm, Stem Cell and Brain Research Institute U1208, 69500 Bron, France.

[2] Inovarion, 75005 Paris, France

[3] La Structure Fédérative de Recherche Santé Lyon-Est, CNRS UAR 3453, INSERM US7, Lyon 1 University, 69008 Lyon, France

[4] Centre d'Etude et de Recherche Multimodal et Pluridisciplinaire en Imagerie du Vivant (CERMEP), 69677 Bron, France

[5] Integrative Multisensory Perception Action & Cognition Team (ImpAct), INSERM U1028, CNRS UMR5292, Lyon Neuroscience Research Center (CRNL); Université Lyon 1, 69500, Bron, France.

* These authors contributed equally

† **Corresponding authors: Camille Giacometti**, camille.giacometti@inserm.fr; **Fadila Hadj-Bouziane,** fadila.hadj-bouziane@inserm.fr; **Céline Amiez**. INSERM U1208, Stem Cell and Brain Research Institute, 69500 Bron, France. E-mail address: celine.amiez@inserm.fr.

This PDF file includes:

Supplementary tables:

S1: GLMM statistical results
S2: SEEDs/ROIs xoxel size and volume in Human
S3: SEEDs/ROIs Voxel size and volume in Macaque

Supplementary figures:

S1: Figure 2 equivalent for the left hemisphere
S2: Figure 3 equivalent for the left hemisphere
S3: Results of statistical T.test Z-scores comparison to 0
S4: SEEDs-ROIs Euclidean distance in the right hemisphere
S5: SEEDs-ROIs Euclidean distance in the left hemisphere
S6: SEEDs-ROIs Z-scores correlation with Euclidean distance
S7: Macaques: sleepy session analysis
S8: Macaques: anaesthetized state analysis
S9: Whole amygdala analysis humans, anaesthetized and awake macaques
S10: Results of AMG SEED LA sampling in human
S11: Human: ROIs position in the brain with a paracingulate sulcus

Supplementary Tables

	Humans	
	**Right hemisphere**	**Left hemisphere**
**SEEDS**	F(3,1197)=19.103, p=4.197e-12	F(3,1193)= 8.2708, p = 1.901e-05
**ROIs**	F(15,1197)=28.805, p<2.2e-16	F(15, 1193)= 22.8448, p< 2.2e-16
**SEEDS*ROIs**	F(45, 1197)=2.782, p=6.531e-09	F(45, 1193)= 1.6543, p=0.004638
	**Macaques Rhesus**	
	**Right hemisphere**	**Left hemisphere**
**SEEDS**	F(3, 2205)=0.8367, p=0.47364	F(3, 2205)= 4.2813, p=0.005067
**ROIs**	F(15, 2205)=22.5703, p<2e-16	F(15, 2205)= 8.3530, p< 2.2e-16
**SEEDS*ROIs**	F(45, 2205)=1.2860, p=0.09731	F(45, 2205)= 1.6695, p=0.003632

Table S1. GLMM statistical results in humans and macaques in the left and right hemispheres.

	HUMAN			
	Left Hemisphere		Right Hemisphere	
**Region**	**Voxel Number**	**Volume (mm3)**	**Voxel Number**	**Volume (mm3)**
**LA**	27±0	421.88±0	23±0	359.38±0
**BL**	22±0	343.75±0	18±0	281.25±0
**BM**	8±0	125±0	7±0	109.38±0
**CE**	3±0	46.88±0	5±0	78.12±0
**Area25**	57.9±2.31	904.69±36.17	54.75±3.71	855.47±57.99
**SROSp**	56.95±2.46	889.84±38.43	56.55±2.42	883.59±37.76
**SROSm**	56.5±3.71	882.81±57.91	55.45±4.11	866.41±64.22
**SROSa**	60.15±3.82	939.84±59.61	59.2±3.37	925±52.59
**Fork32**	57.4±3.14	896.88±48.99	58.4±3.32	912.5±51.8
**CgS11**	79.68±26.42	1245.07±412.81	77.5±26.4	1210.94±412.45
**CgS10**	74.9±29.13	1170.31±455.15	73.3±29.34	1145.31±458.39
**CgS9**	71±28.4	1109.38±443.83	82.35±30.38	1286.72±474.61
**CgS8**	76.55±23.54	1196.09±367.82	78.15±26.24	1221.09±409.94
**CgS7**	84±29.89	1312.5±466.99	82.7±29.52	1292.19±461.18
**CgS6**	75.3±27.54	1176.56±430.31	73.2±29.04	1143.75±453.78
**CgS5**	80.85±28.37	1263.28±443.26	71.7±28.2	1120.31±440.69
**CgS4**	76±29.74	1187.5±464.76	68.95±22.86	1077.34±357.19
**CgS3**	73±28.52	1140.62±445.65	62.15±21.27	971.09±332.32
**CgS2**	61.75±19.97	964.84±312.04	58.9±17.18	920.31±268.48
**CgS1**	57.95±2.42	905.47±37.76	61.95±13.46	967.97±210.27

Table S2. **Humans: SEEDS and ROIs masks volume and number of voxels in the left and right hemisphere.**

	MACAQUE			
	Left Hemisphere		Right Hemisphere	
**Region**	**Voxel Number**	**Volume (mm3)**	**Voxel Number**	**Volume (mm3)**
**LA**	8±0	46.66±0	8±0	46.66±0
**BL**	8±1.73	46.66±10.1	9.33±0.58	54.43±3.37
**BM**	9±0	52.49±0	9±0	52.49±0
**CE**	3±0	17.5±0	3±0	17.5±0
**Area25**	12±0	69.98±0	9.33±2.31	54.43±13.47
**SROSp**	10.33±2.08	60.26±12.14	11.33±0.58	66.1±3.37
**SROSm**	11±1.73	64.15±10.1	12±0	69.98±0
**SROSa**	10±1.73	58.32±10.1	11.67±1.15	68.04±6.73
**Fork32**	9.67±1.15	56.38±6.73	12±0	69.98±0
**CgS11**	9.67±1.53	56.38±8.91	11.33±0.58	66.1±3.37
**CgS10**	11±1	64.15±5.83	11.33±0.58	66.1±3.37
**CgS9**	10.33±2.08	60.26±12.14	12.33±0.58	71.93±3.37
**CgS8**	10±0	58.32±0	10.67±1.15	62.21±6.73
**CgS7**	8.67±1.15	50.54±6.73	11.33±1.15	66.1±6.73
**CgS6**	11±1	64.15±5.83	11.67±1.53	68.04±8.91
**CgS5**	10.67±1.15	62.21±6.73	12±0	69.98±0
**CgS4**	10±1	58.32±5.83	9.67±1.53	56.38±8.91
**CgS3**	10.33±0.58	60.26±3.37	10.67±0.58	62.21±3.37
**CgS2**	9.33±0.58	54.43±3.37	10±1.73	58.32±10.1
**CgS1**	10.33±0.58	60.26±3.37	11±1	64.15±5.83

Table S3. **Macaques: SEEDS and ROIs masks volume and number of voxels in the left and right hemisphere.**

Supplementary Figures

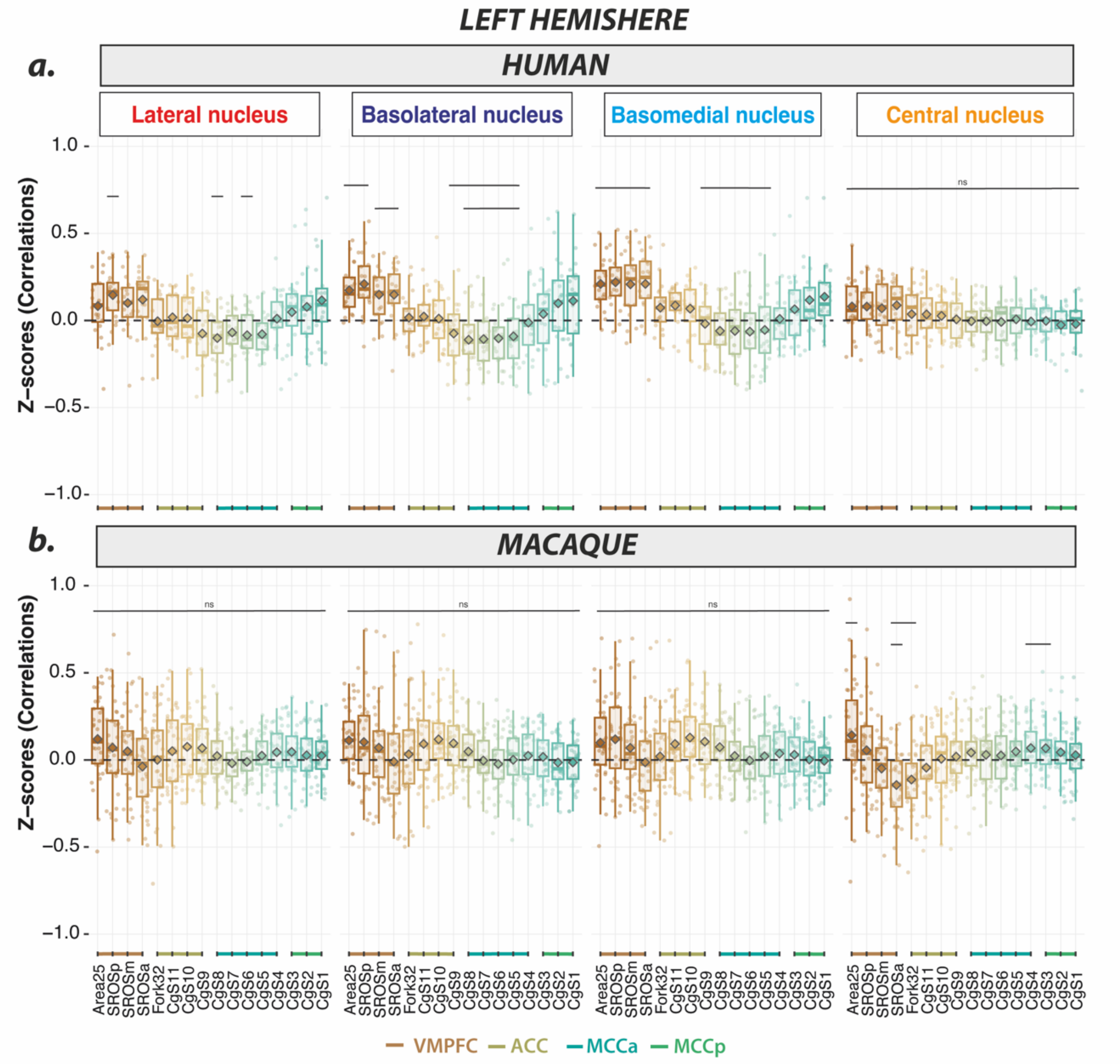

Figure S1. Left hemisphere: Functional connectivity gradient between AMG nuclei and mPFC network in humans and macaques. Boxplots display correlation strength (z-scores) between each AMG nuclei (seed) and the mPFC ROIs for each species. In each plot, the lower and upper hinges of the box correspond to the first and third quartiles respectively. Upper and lower whiskers extent from their corresponding hinges to the largest and lowest value respectively define as 1.5 x of the interquartile range. Diamonds overlaid in dark grey on the boxplots represent the mean. Individual data points are also represented for each boxplot. The horizontal black lines represent significant pairwise results within seeds associated with FDR corrections. **a.** Human: GLMM showed a main effect of SEEDS ($F(3, 1193) = 8.2708$, $p = 1.901e-05$), a main effect of ROIs ($F(15, 1193) = 22.8448$, $p < 2.2e-16$) and an interaction between SEEDs and ROIs ($F(45, 1193) = 1.6543$, $p = 0.004638$). Pairwise significant comparison p.value range from 1.157737e-05 to 0.04846282 with df=1193. **b.** Macaque: GLMM showed a main effect of SEEDS ($F(3, 2205) = 4.2813$, $p = 0.005067$), a main effect of ROIs ($F(15, 2205) = 8.3530$, $p < 2.2e-16$) and an interaction between SEEDs and ROIs ($F(45, 2205) = 1.6695$, $p = 0.003632$). Pairwise significant comparison p.value range from 3.226877e-05 to 0.04731633 with df= 2205.

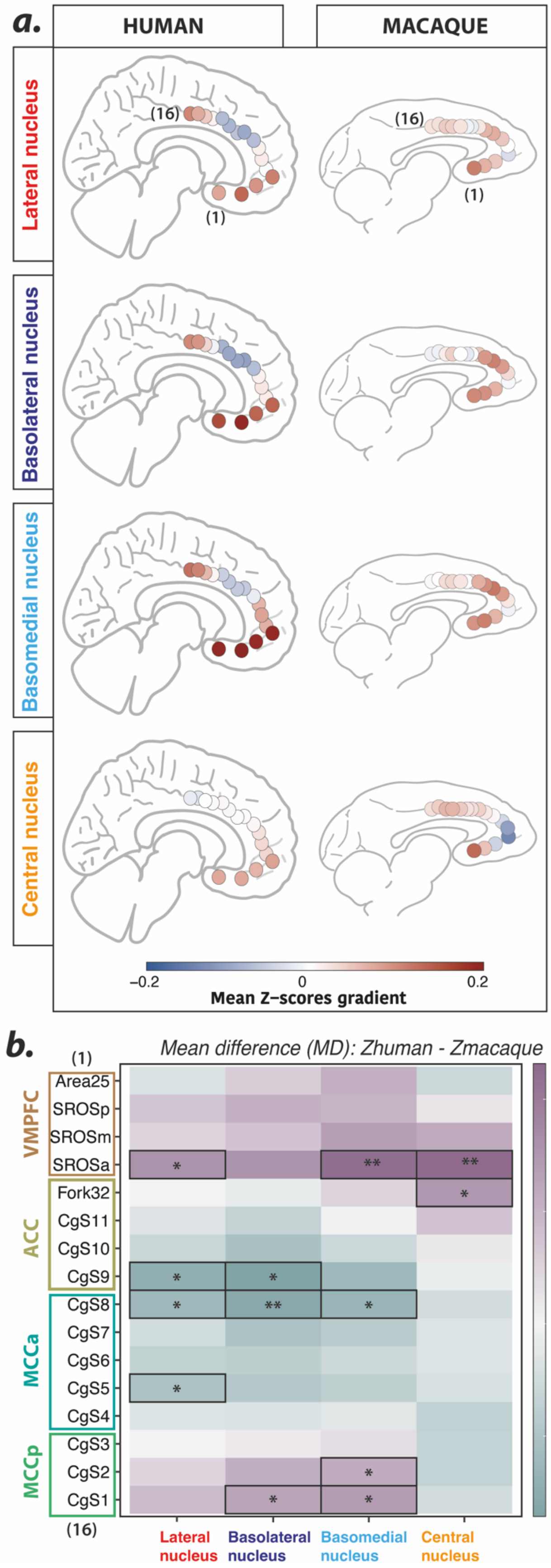

Figure S2. Left hemisphere: comparison between human and macaque functional connectivity between AMG nuclei and mPFC ROIs. a. Human: the most negative FC for LA and BL is located in ROI CgS8 (LA: -0.10±0.14; BL: -0.11±0.17) and ROI CgS6 for BM (-0.06±0.19), that is within aMCC. Macaque: the most negative FC for CE, BM and LA is located in ROI SROSa located in vmPFC (CE: -0.15±0.20; BM: -0.01±0.21; LA: -0.04±0.23), in ROI CgS6 for BL (-0.02±0.16). **b.** The heatmap mean difference show the gradient shift between human and macaque: t.test comparisons show significant differences in MCCa (CgS9 and CgS8) for LA, BL and BM, as well as in vmPFC at the SROS level for LA, BM and CE. Two-sides Student test significant differences between species are highlighted: * for p.value < 0.05 and ** for p.value <0.01 ranging from 0.0017152 to 0.029376.

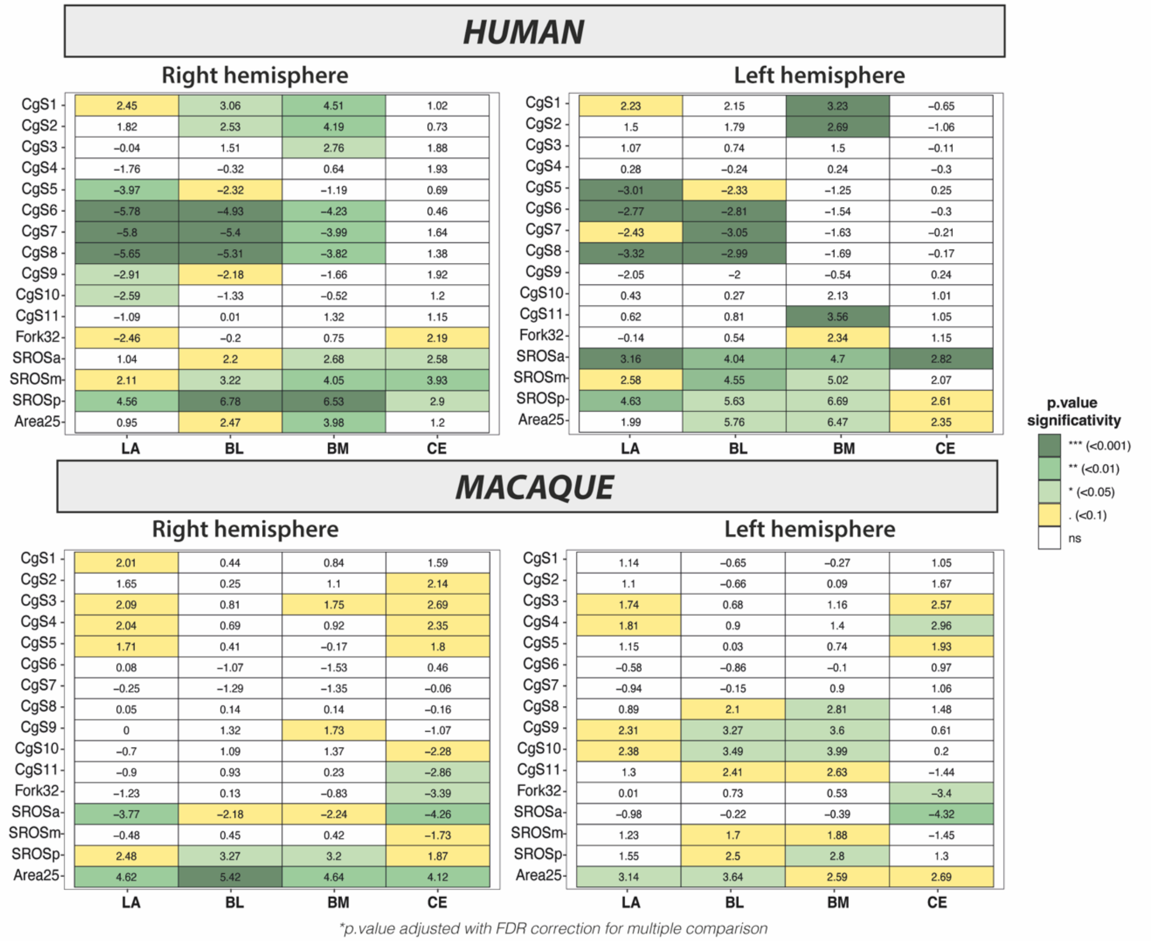

Figure S3. Z-scores comparison to 0. Using a one-sided Student test, each SEED-ROI pair correlation strength (i.e., functional connectivity) is compared to 0 in both hemispheres in humans (top) and macaques (bottom). T.test statistics are displayed in each corresponding tiles. In human, n=20, df=19, in the right hemisphere p.value range from 0.00009568 to 0.04835556 and from 0.00010688 to 0.04608000 in the left hemisphere. In macaques, n=36 (3*12 runs), df= 35, p.value ranges from 0.000288 to 0.045504 in the right hemisphere and from 0.00774400 to 0.04788364 in the left hemisphere.

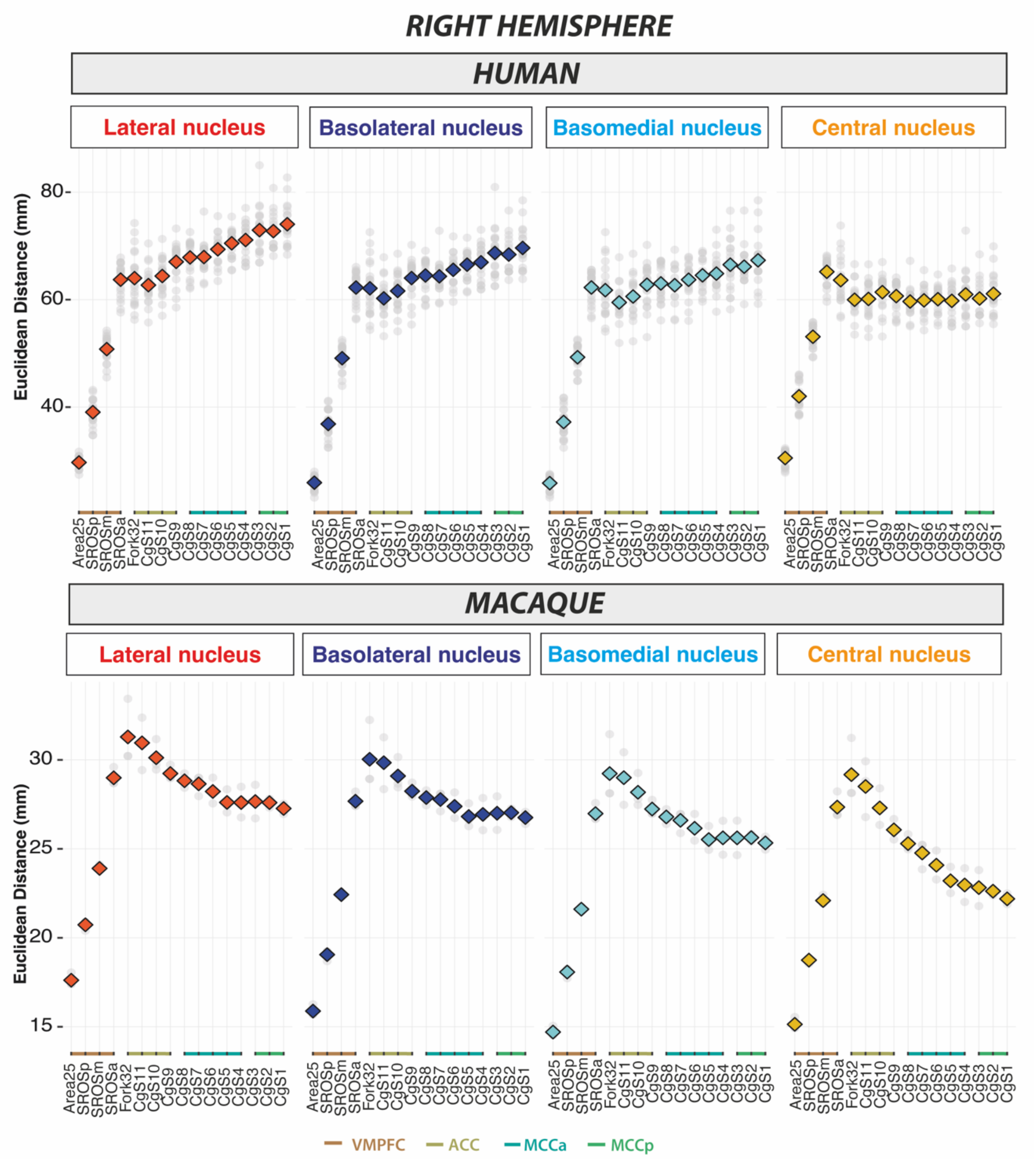

Figure S4. Right hemisphere Euclidean distance (in mm) between AMG nuclei and mPFC ROIs in humans (top) and macaques (bottom).

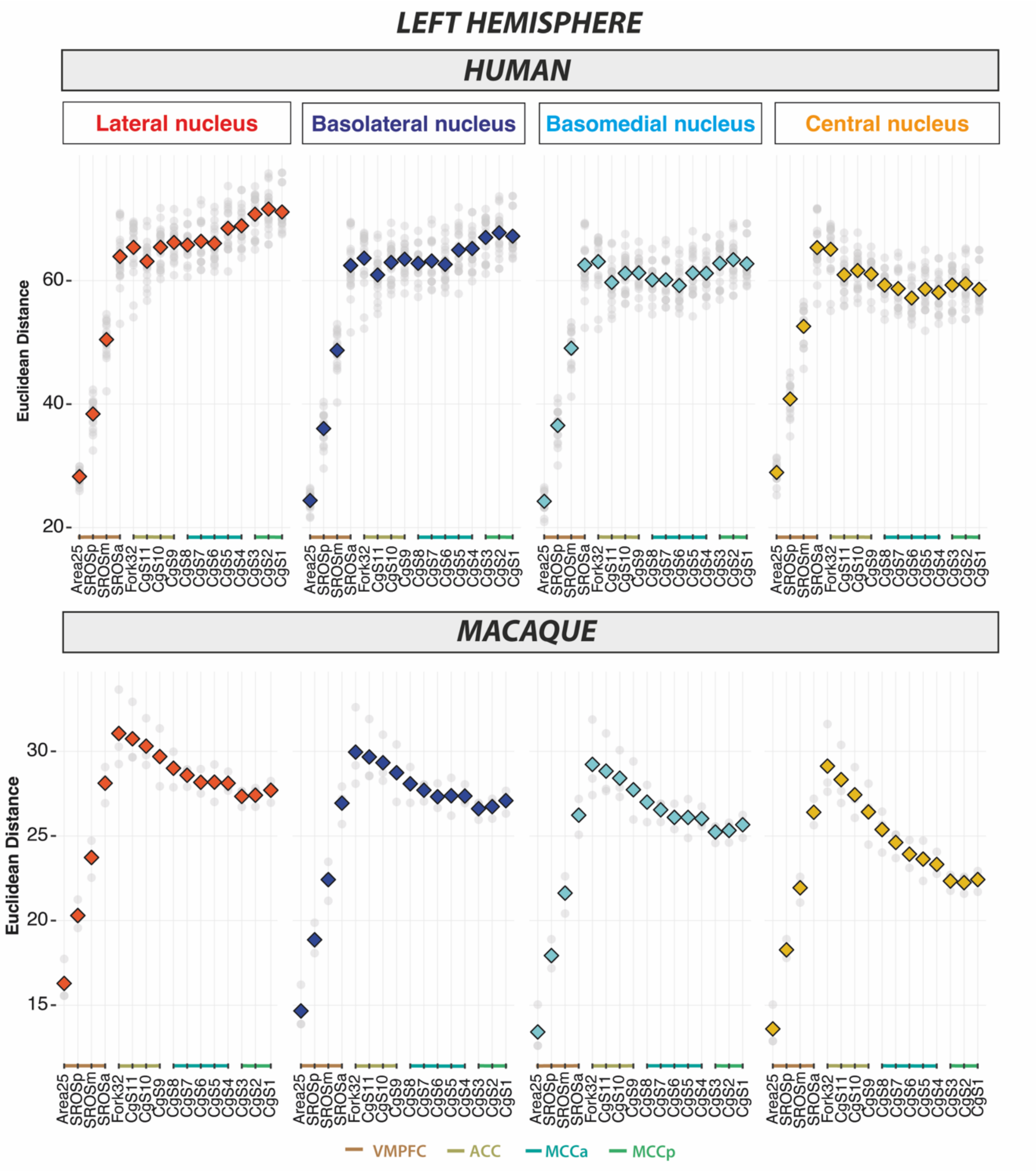

Figure S5. Left hemisphere Euclidean distance (in mm) between AMG nuclei and mPFC ROIs humans (top) and macaques (bottom).

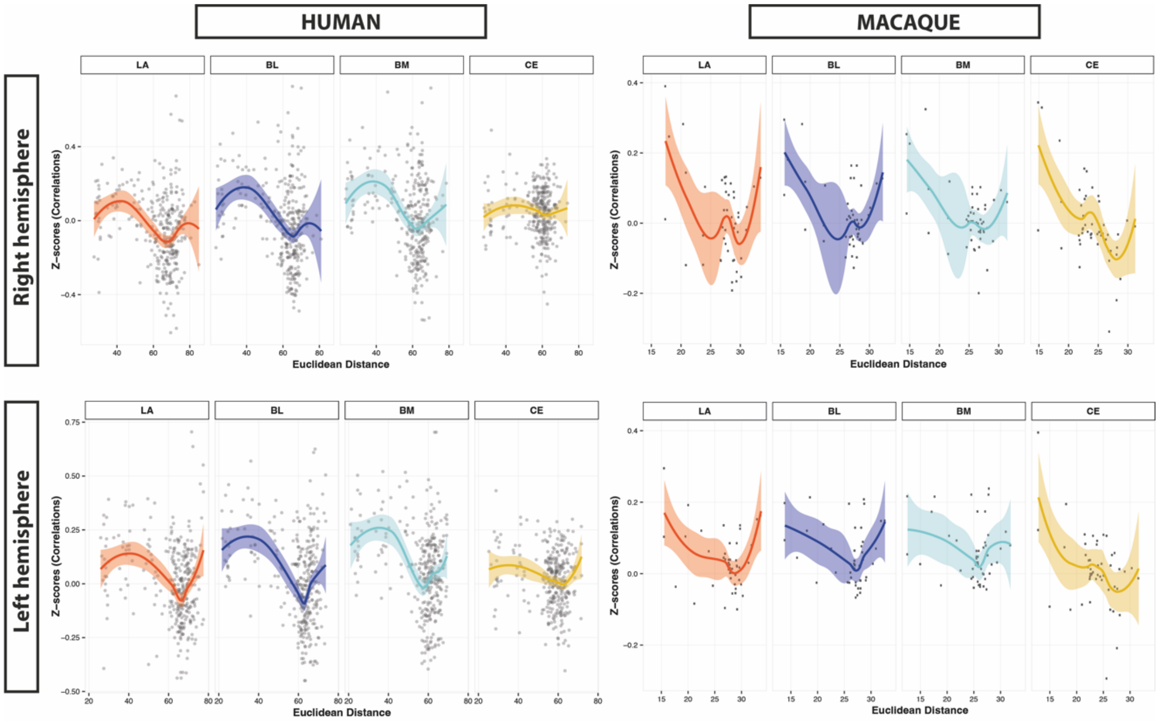

Figure S6. Z-scores expressed as a function of the Euclidean distance in humans and macaques. Plots represent the correlation -z-scores- between each seed and ROIs (y) in correlation with the Euclidean distance (x) for both the right (top panel) and left (bottom panel) hemisphere. Humans and macaques' results are displayed in the left panel and right panel respectively. Results are displayed for each SEED separately: LA= lateral nucleus (red), BL= basolateral nucleus (dark blue), BM= basomedial nucleus (light blue), CE= central nucleus (yellow). Results show that for both species the Euclidean distance does not predict the Z-score values.

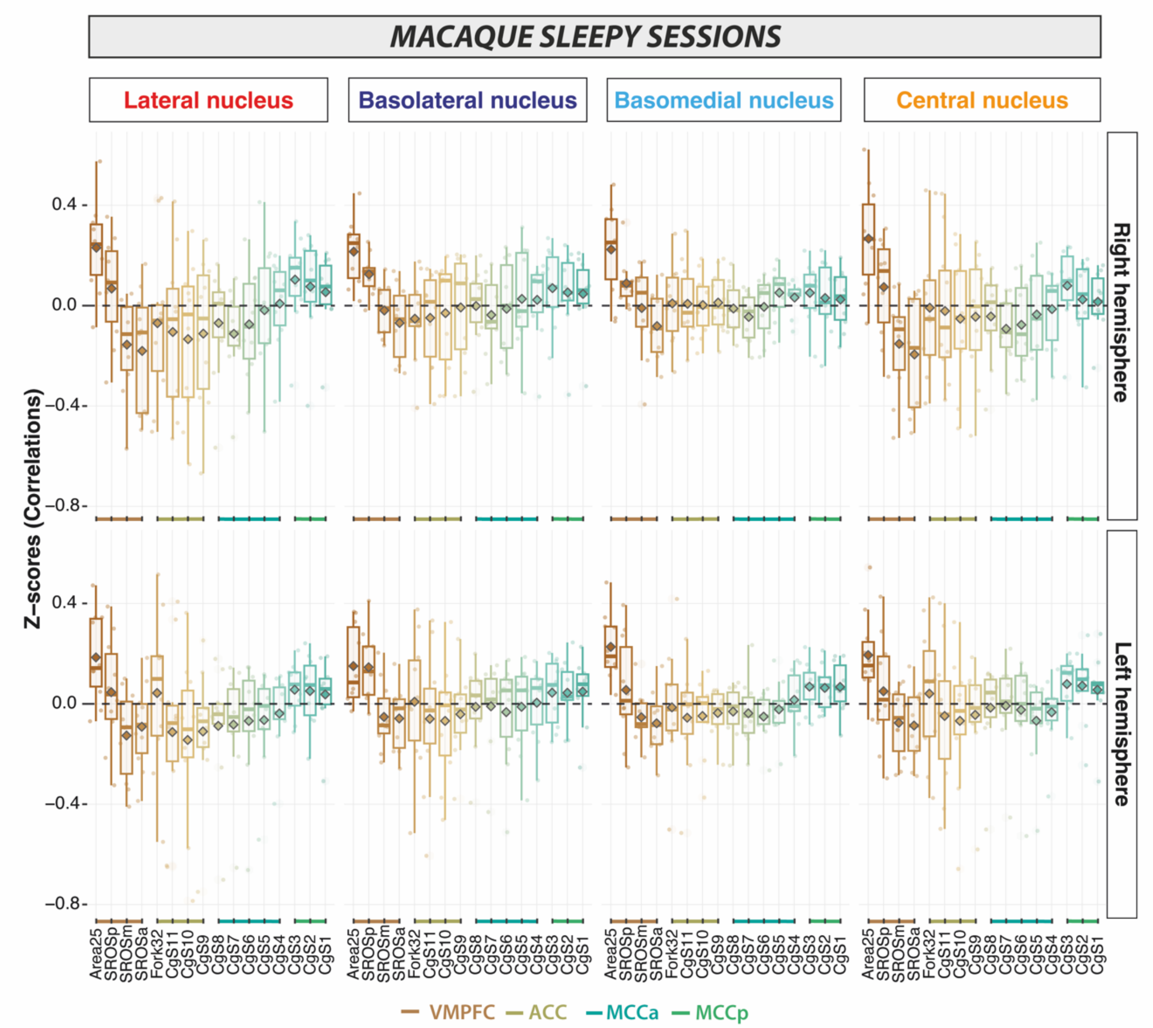

Figure S7. Functional connectivity pattern between AMG nuclei and mPFC ROIs in macaques during sleepy runs, without rewards. Boxplots representing the functional connectivity of AMG nuclei and mPFC ROIs across 2 monkeys (L and N, 6 and 4 runs respectively) in the right and left hemispheres. Results show a similar pattern of the FC fluctuations as in the fixation-reward task presented in the main text Figure 3.

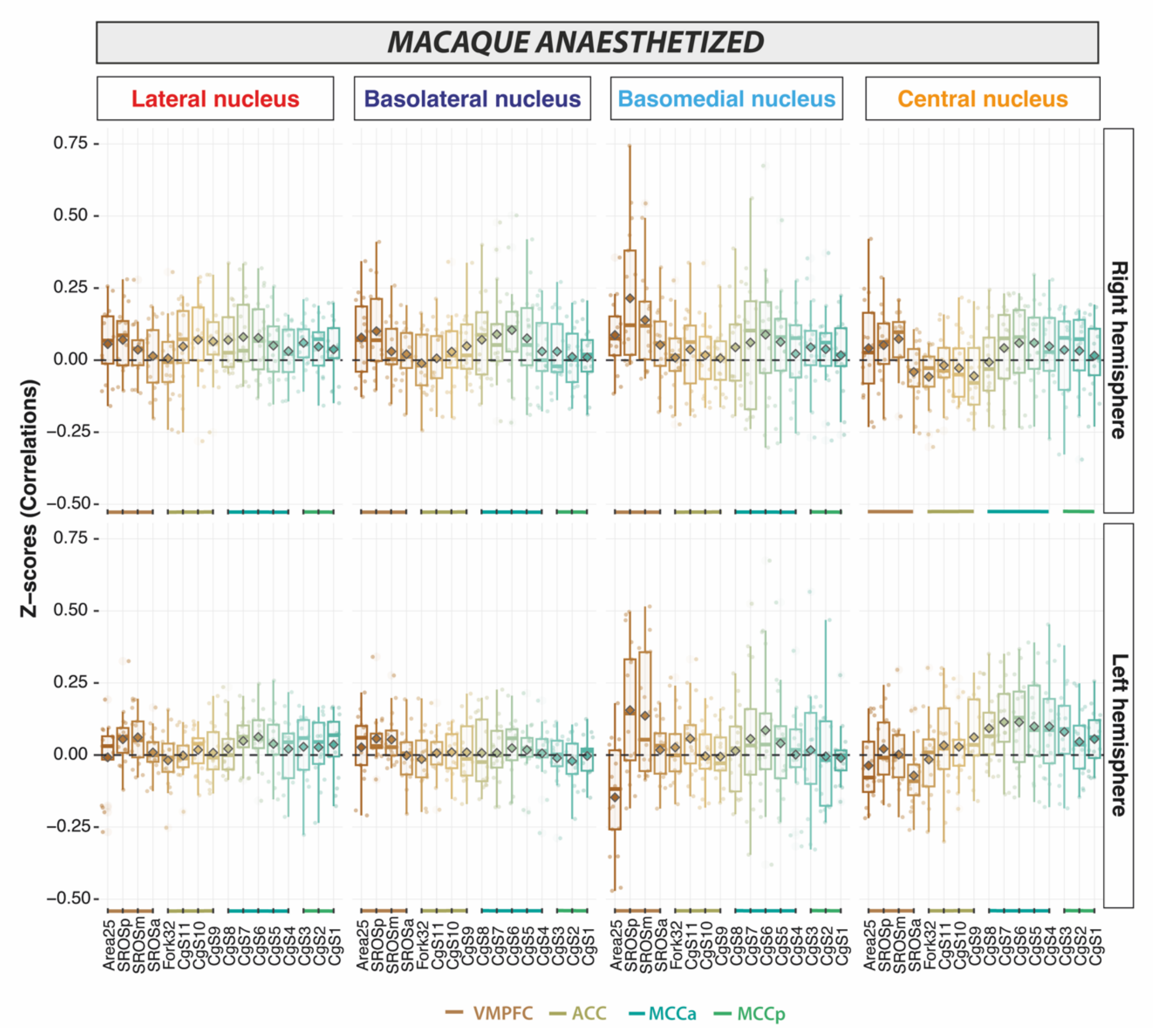

Figure S8. Functional connectivity pattern between AMG nuclei and mPFC ROIs in anaesthetized macaques. Boxplots representing the functional connectivity of AMG nuclei and mPFC ROIs across the 3 monkeys (C, L and N) in the right and left hemispheres under isoflurane (anaesthesia protocol details in Giacometti et al. 2021). Results show a reduction of the FC fluctuations compared to the awake state in the same group of macaque monkeys (Figure 3, main text).

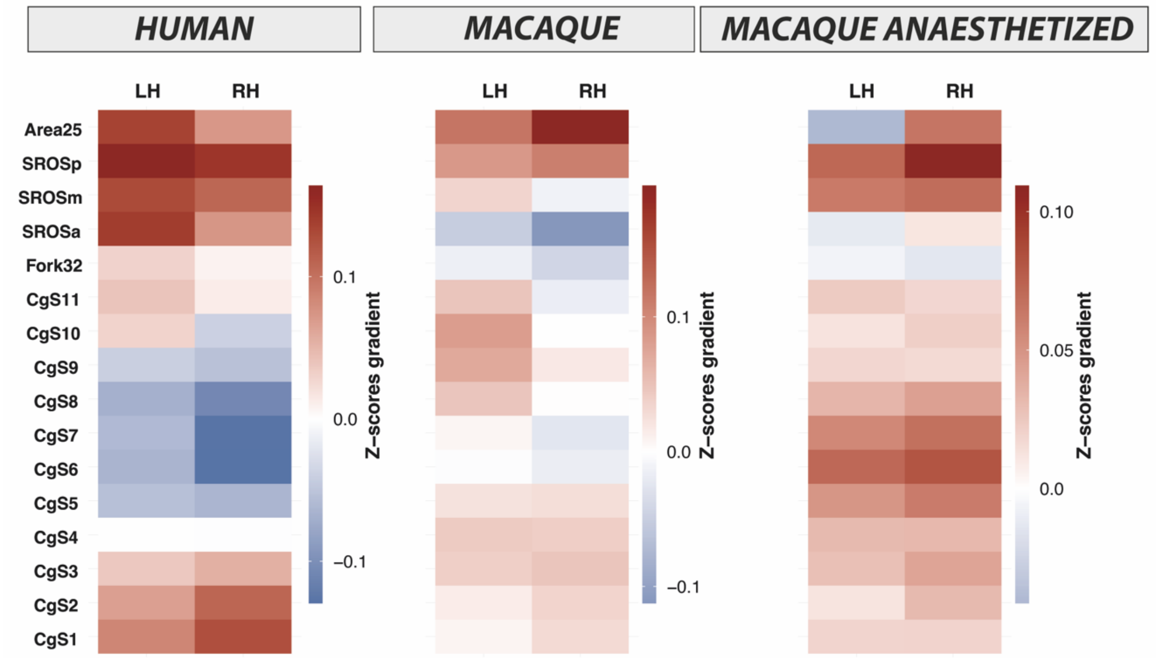

Figure S9. Amygdala functional connectivity with mPFC ROIs in humans, awake and anaesthetized macaques. Heatmap of mean z-scores are represented in both hemispheres. The amygdala here denotes the combination of the 4 AMG Seed masks described above in each species.

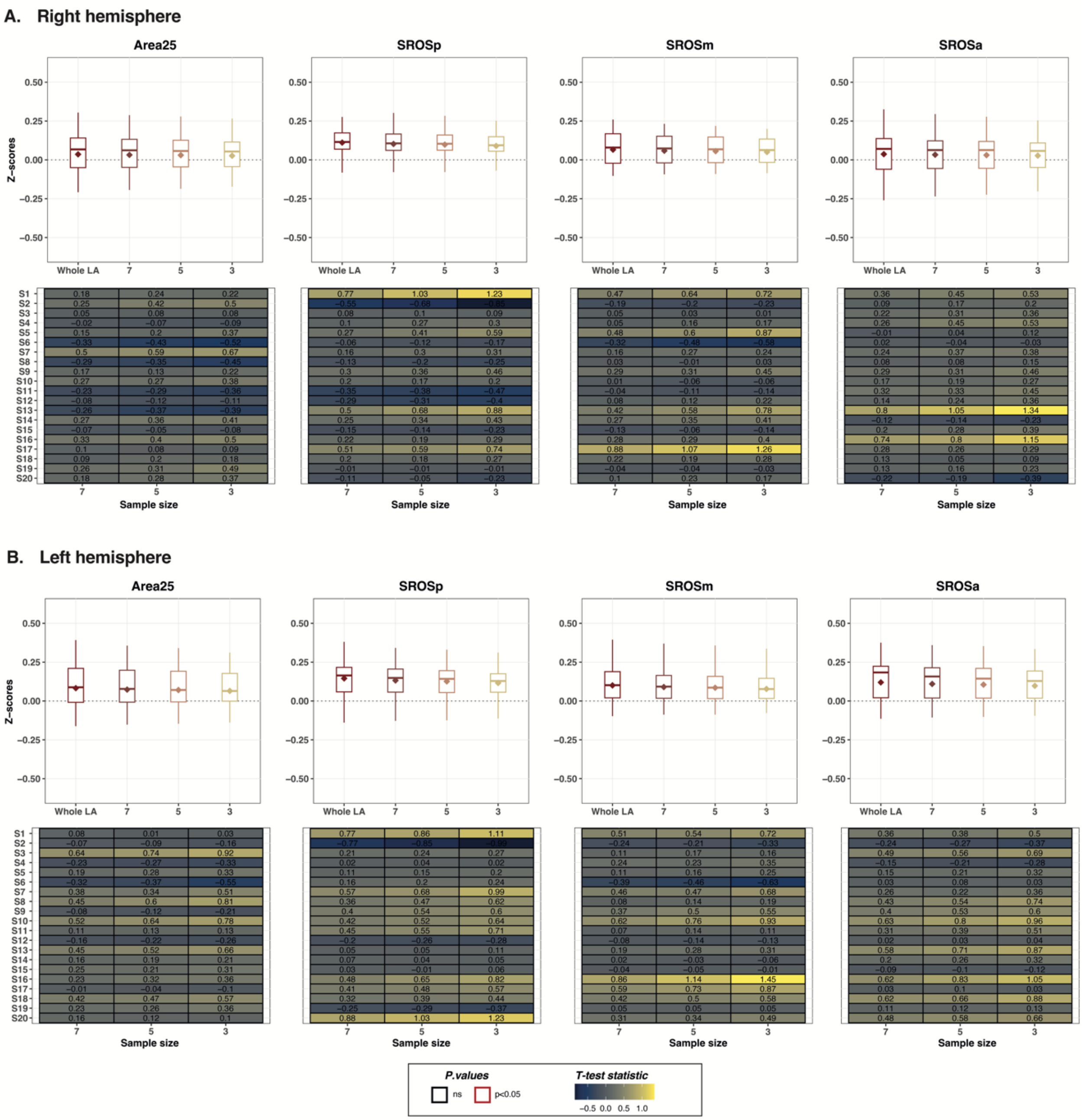

Figure S10. Correlation profile between vmPFC ROIs and different numbers of voxels within the amygdala LA nucleus in humans. (**A**) Right and (**B**) left hemispheres, _Top panels_: correlation strength between 4 vmPFC ROIs (namely: Area25, SROSp, SROSm and SROSa) and either the mean of the whole LA nucleus or the mean of a random subsampling of 7, 5 or 3 voxels among the LA nucleus, obtained using bootstrapping methods. Specifically, for each participant and hemisphere, we randomly and successively sampled 3, 5 and 7 voxels within the LA nucleus (variable termed "Sample_Size" thereafter) without replacement. This sampling was repeated 1000 times for each participant, "Sample_Size" and hemisphere. As for the whole LA nucleus

analysis, the signal was then averaged across voxels for each repetition, “Sample_Size”, hemisphere and participants. We then computed the correlation strength (Z-scores) between each of these averages and the 4 vmPFC ROIs that are displayed on the box-plots as a function of voxels number (whole LA, 7, 5 and 3 voxels). In each plot, the vertical length of the box represents the interquartile range, the thick horizontal line represents the median, and the whiskers indicate the full range of values. Diamonds overlaid on the boxplots represent the mean. *Bottom panels:* one sample t tests performed for each participant to compare the correlation strength derived from the different sampling procedure (3, 5 and 7 voxels) with the mean correlation strength of the whole LA nucleus (reference). Values inside the tiles represented t test statistics, color coded with a gradient from blue to yellow, representing negative to positive values. The results show no statistical differences in neither of the participants (bottom panels in A and B for each hemisphere). Overall, the results show a similar pattern of correlation strength for each vmPFC ROI with either the whole LA nucleus or the different sample sizes tested.

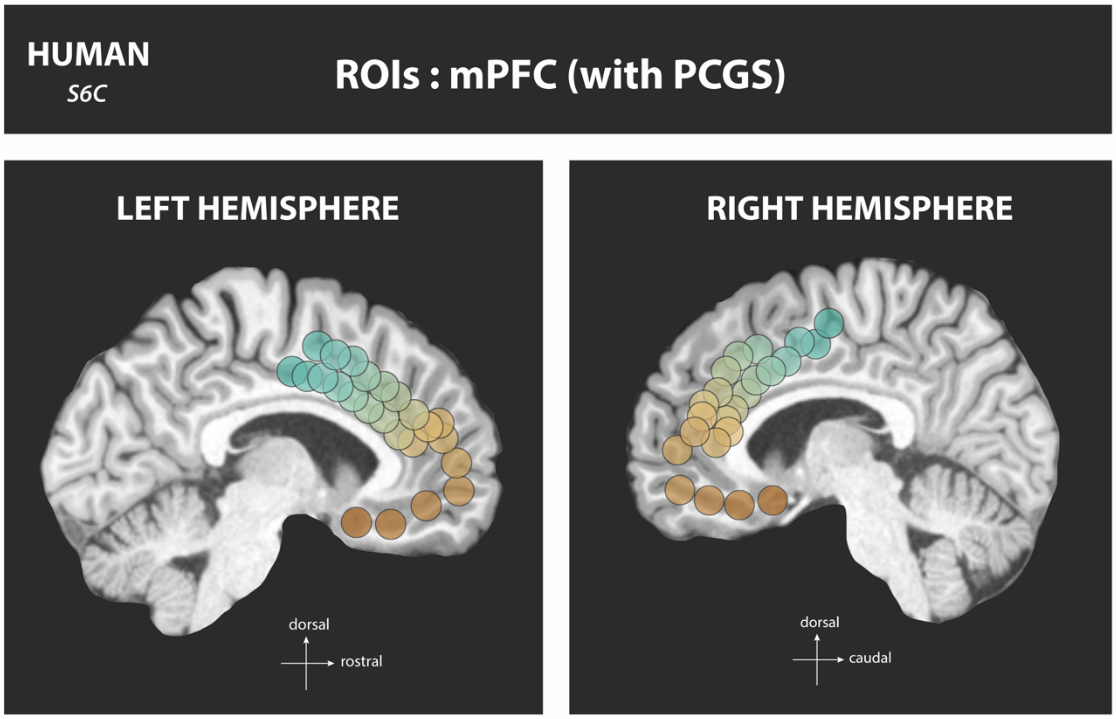

Figure S11. Human: ROIs localization on a subject with a paracingulate sulcus (PCGS) in both hemispheres.
