# Supplementary material for: Differential functional organization of amygdala-medial prefrontal cortex networks in macaque and human
Source: Commun Biol. 2024 Mar 5;7:269. doi: 10.1038/s42003-024-05918-y (PMC10914752; doi:10.1038/s42003-024-05918-y)
Supplement: Supplementary file 2 — Description of Supplementary Materials [file 42003_2024_5918_MOESM2_ESM.docx]

**Description of Additional Supplementary Files**

**File name:** Supplementary Data 1

**Description:** The source data behind the graphs in the article’s main figures.

**File name:** Supplementary Data 2

**Description:** The source data behind the graphs in the article’s main figures.
