# Supplementary material for: Differential functional organization of amygdala-medial prefrontal cortex networks in macaque and human
Source: Commun Biol. 2024 Mar 5;7:269. doi: 10.1038/s42003-024-05918-y (PMC10914752; doi:10.1038/s42003-024-05918-y)
Supplement: Supplementary file 5 — Reporting summary [file 42003_2024_5918_MOESM5_ESM.pdf]

nature portfolio

Corresponding author(s): Giacometti Camille

Last updated by author(s): 01/23/2024

Reporting Summary

Nature Portfolio wishes to improve the reproducibility of the work that we publish. This form provides structure for consistency and transparency in reporting. For further information on Nature Portfolio policies, see our Editorial Policies and the Editorial Policy Checklist.

Statistics

For all statistical analyses, confirm that the following items are present in the figure legend, table legend, main text, or Methods section.

n/a	Confirmed	
☐	☒	The exact sample size (*n*) for each experimental group/condition, given as a discrete number and unit of measurement
☐	☒	A statement on whether measurements were taken from distinct samples or whether the same sample was measured repeatedly
☐	☒	The statistical test(s) used AND whether they are one- or two-sided<br>*Only common tests should be described solely by name; describe more complex techniques in the Methods section.*
☐	☒	A description of all covariates tested
☐	☒	A description of any assumptions or corrections, such as tests of normality and adjustment for multiple comparisons
☐	☒	A full description of the statistical parameters including central tendency (e.g. means) or other basic estimates (e.g. regression coefficient) AND variation (e.g. standard deviation) or associated estimates of uncertainty (e.g. confidence intervals)
☐	☒	For null hypothesis testing, the test statistic (e.g. *F*, *t*, *r*) with confidence intervals, effect sizes, degrees of freedom and *P* value noted<br>*Give P values as exact values whenever suitable.*
☒	☐	For Bayesian analysis, information on the choice of priors and Markov chain Monte Carlo settings
☒	☐	For hierarchical and complex designs, identification of the appropriate level for tests and full reporting of outcomes
☒	☐	Estimates of effect sizes (e.g. Cohen's *d*, Pearson's *r*), indicating how they were calculated

Our web collection on statistics for biologists contains articles on many of the points above.

Software and code

Policy information about availability of computer code

Data collection	Will be available upon publication with the doi access for Figshare depository.
Data analysis	For the MRI images preprocessing, we used SPM12 (Matlab toolbox), FSL and AFNI.<br>For the data processing we mostly used R.

For manuscripts utilizing custom algorithms or software that are central to the research but not yet described in published literature, software must be made available to editors and reviewers. We strongly encourage code deposition in a community repository (e.g. GitHub). See the Nature Portfolio guidelines for submitting code & software for further information.

Data

Policy information about availability of data

All manuscripts must include a data availability statement. This statement should provide the following information, where applicable:

- Accession codes, unique identifiers, or web links for publicly available datasets
- A description of any restrictions on data availability
- For clinical datasets or third party data, please ensure that the statement adheres to our policy

The authors declare that the data supporting the findings of this study are available within its supplementary information files.

Human research participants

Policy information about [studies involving human research participants and Sex and Gender in Research.](#)

Reporting on sex and gender	Twenty healthy subjects participated in the resting-state fMRI experiment (sex: 14 F and 6 M; age 25.6 ± 5.3) and received a monetary compensation at the end of the session. The study was approved by a national ethics committee in biomedical research (Comité de Protection des Personnes (CPP) Sud-Est III, authorization ID: 2015-A00897-42 and 2018-A00405-50). It also received Clinical Trial Numbers (NCT03119909 and NCT03483233, see https://clinicaltrials.gov).
Population characteristics	See above.
Recruitment	In person and Online trough advertisement on university and neurosciences participants recruitment page.
Ethics oversight	Comité de Protection des Personnes (CPP), Sud-Est III

Note that full information on the approval of the study protocol must also be provided in the manuscript.

Field-specific reporting

Please select the one below that is the best fit for your research. If you are not sure, read the appropriate sections before making your selection.

☒ Life sciences ☐ Behavioural & social sciences ☐ Ecological, evolutionary & environmental sciences

For a reference copy of the document with all sections, see nature.com/documents/nr-reporting-summary-flat.pdf

Life sciences study design

All studies must disclose on these points even when the disclosure is negative.

Sample size	20 in humans and 3 in macaques
Data exclusions	NA
Replication	NA
Randomization	Not needed for resting-state analysis, as no behavioral date were acquired.
Blinding	NA

Reporting for specific materials, systems and methods

We require information from authors about some types of materials, experimental systems and methods used in many studies. Here, indicate whether each material, system or method listed is relevant to your study. If you are not sure if a list item applies to your research, read the appropriate section before selecting a response.

Materials & experimental systems

n/a	Involved in the study	
☒	☐	Antibodies
☒	☐	Eukaryotic cell lines
☒	☐	Palaeontology and archaeology
☐	☒	Animals and other organisms
☒	☐	Clinical data
☒	☐	Dual use research of concern

Methods

n/a	Involved in the study	
☒	☐	ChIP-seq
☒	☐	Flow cytometry
☐	☒	MRI-based neuroimaging

Animals and other research organisms

Policy information about [studies involving animals](#); [ARRIVE guidelines](#) recommended for reporting animal research, and [Sex and Gender in Research](#)

Laboratory animals	Three rhesus monkeys (Macaca mulatta) were included in the study

Laboratory animals	(2 F: Monkeys C, 21 yo and N 9.5 yo and 1 M: Monkey L, 9.5 yo; weight 5 - 8 kg).
Wild animals	NA
Reporting on sex	NA
Field-collected samples	NA
Ethics oversight	French Animal Experimentation Ethics Committee #42 (CELYNE)

Note that full information on the approval of the study protocol must also be provided in the manuscript.

Magnetic resonance imaging

Experimental design

Design type	Resting-state
Design specifications	1 run of 10min in humans;<br>12 runs of 10min in non-human primates
Behavioral performance measures	/

Acquisition

Imaging type(s)	functional and structural
Field strength	3T
Sequence & imaging parameters	EPI (T2* weighted) and T1 MPRAGE in both species. With multiband and multiecho in humans.
Area of acquisition	Whole brain

Diffusion MRI ☐ Used ☒ Not used

Preprocessing

Preprocessing software	SPM12, AFNI and FSL
Normalization	Normalized to each species respective templates.
Normalization template	MNI for humans and NMT v2 for macaques.
Noise and artifact removal	Motion parameters and WM/CSF
Volume censoring	5 first volume removed to account for signal stabilization.

Statistical modeling & inference

Model type and settings	Linear mixed models with fixed and random effects, Student Tests.
Effect(s) tested	Linear Mixed Model were used for the statistical analysis to decipher the similarity and differences of Seed-ROI pairs.

Specify type of analysis: ☐ Whole brain ☒ ROI-based ☐ Both

Anatomical location(s): Manual location and probabilistic atlas were used.

Statistic type for inference<br>(See Eklund et al. 2016)	NA
Correction	FDR corrected for multiple comparisons.

Models & analysis

n/a	Involved in the study
☐	☒ Functional and/or effective connectivity
☒	☐ Graph analysis
☒	☐ Multivariate modeling or predictive analysis

Functional and/or effective connectivity: Pearson correlations with Fisher normalization (i.e., z-scores).
